# Premature aging of skeletal stem/progenitor cells rather than osteoblasts causes bone loss with decreased mechanosensation

**DOI:** 10.1038/s41413-023-00269-6

**Published:** 2023-07-05

**Authors:** Ruici Yang, Dandan Cao, Jinlong Suo, Lingli Zhang, Chunyang Mo, Miaomiao Wang, Ningning Niu, Rui Yue, Weiguo Zou

**Affiliations:** 1 State Key Laboratory of Cell Biology, Shanghai Institute of Biochemistry and Cell Biology, Center for Excellence in Molecular Cell Science, Chinese Academy of Sciences, University of Chinese Academy of Sciences, Shanghai, 200031 China; 2 Shenzhen Institute of Synthetic Biology, Shenzhen Institute of Advanced Technology, Chinese Academy of Sciences, Shenzhen, 518055 China; 3 Institute for Regenerative Medicine, Shanghai East Hospital, Shanghai Key Laboratory of Signaling and Disease Research, Frontier Science Center for Stem Cell Research, School of Life Sciences and Technology, Tongji University, Shanghai, 200092 China; 4 Institute of Microsurgery on Extremities, and Department of Orthopedic Surgery, Shanghai Sixth People’s Hospital Affiliated to Shanghai Jiao Tong University School of Medicine, Shanghai, 200233 China

**Keywords:** Bone, Bone quality and biomechanics

## Abstract

A distinct population of skeletal stem/progenitor cells (SSPCs) has been identified that is indispensable for the maintenance and remodeling of the adult skeleton. However, the cell types that are responsible for age-related bone loss and the characteristic changes in these cells during aging remain to be determined. Here, we established models of premature aging by conditional depletion of *Zmpste24* (*Z24*) in mice and found that *Prx1-*dependent *Z24* deletion, but not *Osx-*dependent *Z24* deletion, caused significant bone loss. However, *Acan*-associated *Z24* depletion caused only trabecular bone loss. Single-cell RNA sequencing (scRNA-seq) revealed that two populations of SSPCs, one that differentiates into trabecular bone cells and another that differentiates into cortical bone cells, were significantly decreased in *Prx1-Cre; Z24*^*f/f*^ mice. Both premature SSPC populations exhibited apoptotic signaling pathway activation and decreased mechanosensation. Physical exercise reversed the effects of *Z24* depletion on cellular apoptosis, extracellular matrix expression and bone mass. This study identified two populations of SSPCs that are responsible for premature aging-related bone loss. The impairment of mechanosensation in *Z24*-deficient SSPCs provides new insight into how physical exercise can be used to prevent bone aging.

## Introduction

Bone is a dynamic tissue that undergoes a lifelong, highly regulated remodeling process that maintains a balance between bone resorption and formation.^1^ Degenerative bone loss is associated with decreased numbers of osteoblasts and increased numbers of osteoclasts.^2^ Medical treatments for osteoporosis include systemic anabolic medications that enhance osteoblast capacity and anticatabolic medications that decrease osteoclast resorption.^1^ Current drugs are effective in improving bone quality but have side effects.^3,4^ Aging-related changes that occur in skeletal stem/progenitor cells (SSPCs) that form mature osteogenic cells, such as osteoblasts, remain to be further elucidated in order to prevent age-related bone loss.

Different SSPC subsets are identified by the expression of different cell markers, such as paired related homeobox 1 (*Prx1*, also called *Prrx1*) and Osterix (*Osx*, also called *Sp7*). Prx1 plays an essential role in regulating skeletal development in limb buds and adult skeletal stem cells that are derived from the lateral plate mesoderm.^5,6^ Fetal perichondrium-resident *Osx*-expressing cells have been reported to translocate into the nascent marrow cavity, giving rise to new bone tissue and long-lived bone marrow stroma.^7^ *Osx* is also expressed by prehypertrophic and hypertrophic chondrocytes,^8^ periosteal cells, trabecular osteoblasts and osteocytes. However, the types of skeletal cells that are responsible for age-related bone loss remain to be identified.

Hutchinson-Gilford progeria syndrome (HGPS) is a human disease that is characterized by premature aging and significantly affects the skeletal system.^9^ This rare disease is caused by progerin, a truncated form of nuclear Lamin A (a type of intermediate filament). Lamin A forms the nuclear lamina scaffold that is adjacent to the inner nuclear membrane and confers viscosity and stiffness to nuclei.^10^ FACE1, a human metalloproteinase, and its homolog Zmpste24 (Z24) in mice are indispensable for the maturation of Lamin A from its precursor (Prelamin A).^11,12^ Both Lamin A mutations^13,14^ and Z24 deficiency^15,16^ are associated with human diseases that are characterized by severe defects in nuclear stability, cytoskeletal dynamics and nucleocytoskeletal force transmission. In terms of mouse phenotype, cortical and trabecular bone volumes are significantly reduced in Z24-knockout mice.^11,17^ However, whether Z24 specifically functions in bone mesenchymal stem cells has not been elucidated. The generation of conditional Z24-knockout mice could facilitate the elucidation of the specific function of Z24 in the bone system. More importantly, the identification of vital cell populations that are responsible for bone loss due to Z24 deletion has not been achieved.

In this study, we established a model of premature aging via the conditional deletion of Z24 using *Prx1*-*Cre* and *Osx-Cre* mice. The loss of bone in *Prx1*^*Cre*^*;Z24*^*fl/fl*^ mice rather than in *Osx*^*Cre*^*;Z24*^*fl/fl*^ mice prompted us to identify the cell populations that are responsible for premature age-related bone loss. It is well known that Osx-expressing cells include prehypertrophic and hypertrophic chondrocytes, periosteal cells and osteoblasts.^18^ However, to our surprise, the premature aging of these cell populations did not lead to bone loss. By single-cell RNA sequencing (scRNA-seq), we identified two skeletal stem/progenitor cell (SSPC) populations that were enriched in *Prx1*^*Cre*^ mice compared to *Osx* ^*Cre*^ mice. Moreover, these two cell populations were decreased in *Prx1*^*Cre*^*;Z24*^*fl/fl*^ mice compared to their control littermates, suggesting that the premature aging of these two populations was responsible for age-related bone loss. Interestingly, we found that SSPCs that resided in the growth plate (gpSSPCs) maintain only trabecular bone according to the phenotype of *Acan*^*Cre*^*; Z24*^*fl/fl*^ mice. Chromatin and transcriptional profiling demonstrated that prematurely aged SSPCs exhibited apoptotic signaling pathway activation and decreased mechanosensation of compression or tension. Furthermore, we found that mechanical stimulation increased the number of SSPCs (CD73^+^) and reversed the effects of Z24 depletion on cellular apoptosis and bone mass.

## Results

### *Prx1*-dependent *Z24* deficiency, not *Osx*-dependent *Z24* deficiency, causes a premature skeletal phenotype

To establish conditional models of premature aging, we first obtained *Z24-KO* first (Conditional Ready) mice (Fig. S1a). We verified the *Z24* knockout efficiency in different tissues by qPCR (Fig. S1b). As expected, the absence of Z24 resulted in the accumulation of Prelamin A (Fig. S1c). Consistent with the fact that HGPS patients have normal weights at birth,^9^ *Z24-KO* first mice did not show a significant decrease in weight compared to littermate controls until 6 weeks of age (Fig. S1d). *Z24-KO* first mice displayed normal skeletal development at birth, as revealed by whole-mount staining of skeletal samples from postnatal day 1 mice (Fig. S1e–g). *Z24-KO* first mice also displayed normal growth plates at postnatal day 3 according to Safranin O staining (Fig. S1h). Microcomputer tomography (microCT) analysis revealed that *Z24-KO* first mice displayed significant bone loss compared to littermate controls, including decreased bone volume fraction (BV/TV), trabecular thickness (Tb.Th), trabecular number (Tb.N), cortical thickness (Ct.Th) and increased trabecular spacing (Tb.Sp) (Fig. S1i–n). However, tartrate-resistant acid phosphatase (TRAP) staining demonstrated that the number of osteoclasts in the trabecular bones of *Z24-KO* first mice did not significantly differ from their littermate controls (Fig. S1o, p). The phenotypes of our *Z24-KO* first mice were similar to those of two other reported *Z24*-knockout mouse strains,^11,17,19^ although the premature phenotype of our *Z24-KO* first mice appeared later.

**Fig. 1:**
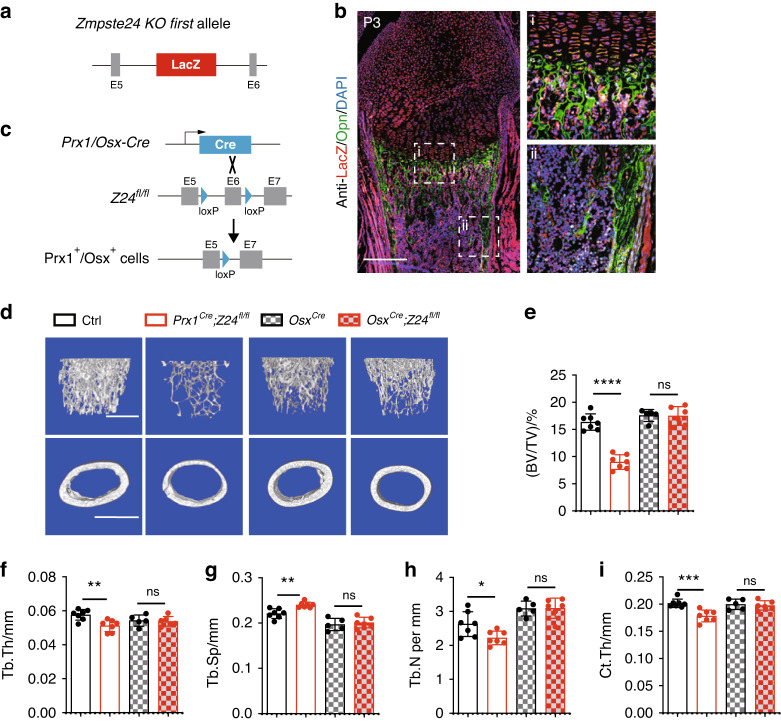
*Prx1*^*Cre*^*;Z24*^*fl/fl*^ mice exhibit age-dependent bone loss, but *Osx*^*Cre*^*;Z24*^*fl/fl*^ mice do not. **a** Schematic showing genetic design of the *Zmpste24* (*Z24*) *KO first* allele. **b** Immunofluorescence image of LacZ and Opn in the femurs of *Z24-LacZ* reporter mice on postnatal Day 3. Scale bar, 3 mm. **c** Schematic diagram showing the strategy for *Z24* deletion (mutant) in Prx1^+^ and Osx^+^ cells. Prx1/Osx-dependent Cre recombinase expression leads to recombination between the loxP sites flanking exon 6 (E6) of the *Z24*^*fl/fl*^ gene. The resulting mutant *Z24* is nonfunctional. **d** Representative microCT images of femurs. Scale bar, 1 mm. Ctrl: Control, Ctrl as the combination of *Z24*^*fl/fl*^ and *Prx1-Cre* mice. Quantitative analyses of trabecular bone volume/total volume (BV/TV) (**e**), trabecular thickness (Tb.Th) (**f**), trabecular spacing (Tb.Sp) (**g**), trabecular number (Tb.N) (**h**) and cortical thickness (Ct.Th) (**i**) in the femurs. The statistical significance of differences was assessed using two-tailed Student’s paired *t* test (*P < 0.05, **P < 0.01, ***P < 0.001 and ****P < 0.000 1)

Taking advantage of the IRES-lacZ sequence that was inserted into the Z24 locus, we found that Z24-expressing cells localized to the cartilage, trabecular bone, and cortical bone on postnatal day 3 (Fig. 1a, b). Thus, we conditionally deleted *Z24* by crossing *Z24*^*fl/fl*^ mice with *Prx1-Cre* or *Osx-Cre* mice to induce premature aging in Prx1-expressing or Osx-expressing skeletal lineage cells, respectively (Fig. 1c, Fig. S2a). Then, we verified the *Z24* knockout efficiency in bone by qPCR (Fig. S2b). Because the *Osx*-*Cre* transgenic mice showed malocclusion, we chose *Osx*^*Cre*^ mice as littermate controls for *Osx*^*Cre*^*;Z24*^*fl/fl*^ mice. Similar to *Z24-KO* first mice, *Prx1*^*Cre*^*;Z24*^*fl/fl*^ mice displayed normal body weights before 12 weeks of age, normal growth plates at p3 and normal skeletal development at p1 (Fig. S2c–f). *Prx1*^*Cre*^*;Z24*^*fl/fl*^ mice also had normal growth plates on postnatal day 3 according to Safranin O staining (Fig. S2g). However, 16-week-old *Prx1*^*Cre*^*;Z24*^*fl/fl*^ mice displayed significantly decreased bone volume fraction, trabecular thickness, trabecular number, and cortical thickness and increased trabecular spacing (Fig. 1d–i). Interestingly, 16-week-old *Osx*^*Cre*^*;Z24*^*fl/fl*^ mice did not display significant changes in bone parameters compared to control mice (Fig. 1d–i). Similarly, tartrate-resistant acid phosphatase (TRAP) staining demonstrated that the number of osteoclasts in the trabecular bones of *Prx1*^*Cre*^*;Z24*^*fl/fl*^ mice did not significantly differ from their littermate controls at both 8 weeks and 16 weeks of age (Fig. S2h, i). Together, these results suggest that Z24 specifically functions in Prx1-expressing cells but not in Osx-expressing cells.

### *Prx1-*dependent *Z24* deficiency leads to a decrease in osteoblast number and bone formation rate

**Fig. 2:**
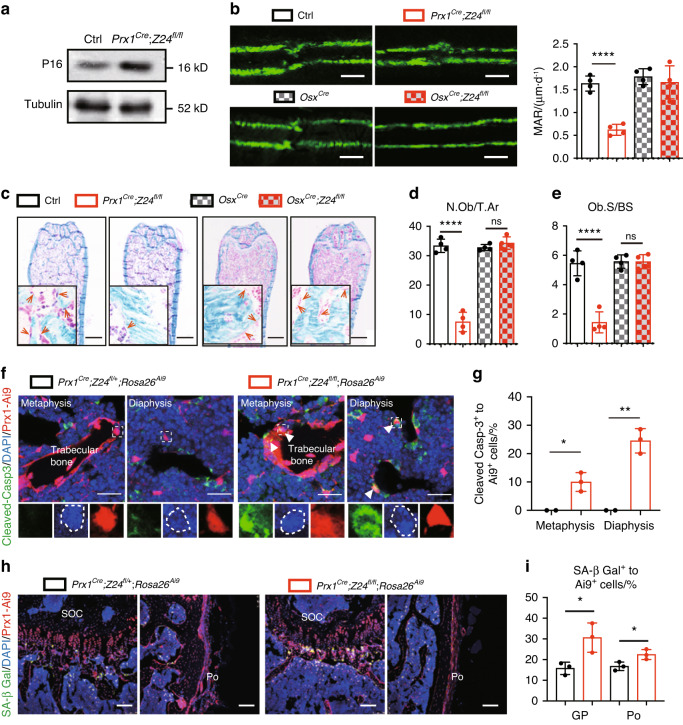
Degeneration of cell renewal in *Prx1*^*Cre*^*;Z24*^*fl/fl*^ mice leads to bone loss. **a** Western blotting analysis of the expression of P16 in bone tissues of *Prx1*^*Cre*^*;Z24*^*fl/fl*^ mice and their littermate controls. Tubulin was used as a reference protein. **b** Calcein double staining and mineral appositional rate (MAR) analysis of 16-week-old *Prx1*^*Cre*^*;Z24*^*fl/fl*^, *Osx*^*Cre*^*;Z24*^*fl/fl*^ and their littermate controls. Scale bars, 20 μm. Data shown are from four replicates. **c** Masson staining of femur hard slides. Scale bars, 80 mm. Quantification of the number of osteoblasts per tissue area (N.Ob/T.Ar) (**d**) and osteoblast surface per bone surface (Ob. BS) (**e**). **f** Immunofluorescence images of cleaved Caspase-3 in the metaphysis and diaphysis. Scale bars, 25 μm. **g** Proportion of cleaved Caspase-3^+^ cells to *Prx1*^*Cre*^*; Rosa26*^*Ai9*^ cells in tissue sections. **h** Immunofluorescence images of SA-β Gal in the growth plate. SOC secondary ossification center, Po periosteum. Scale bars, 100 μm. **i** Proportion of SA-β Gal^+^ cells to *Prx1*^*Cre*^*; Rosa26*^*Ai9*^ cells in tissue sections. The statistical significance of differences was assessed using two-tailed Student’s paired *t* test (*P < 0.05, **P < 0.01, ***P < 0.001 and ****P < 0.000 1)

To further confirm the function of Z24 in Prx1-expressing cells, we examined the expression of the senescence marker P16 in bone tissue. Similar to *KO* first mice, bone tissue of *Prx1*^*Cre*^*;Z24*^*fl/fl*^ mice exhibited elevated protein levels of P16 as shown by Western blotting (Fig. 2a), which indicated the degeneration of bone in *Prx1*^*Cre*^*;Z24*^*fl/fl*^ mice. We next analyzed bone mineralization rates by dynamic histomorphometry analysis, which was performed by calcein double labeling. Compared to their littermate controls, *Prx1*^*Cre*^*;Z24*^*fl/fl*^ mice had a significantly decreased mineral apposition rate (MAR) (Fig. 2b). In contrast, the MAR of *Osx*^*Cre*^*;Z24*^*fl/fl*^ mice was comparable to that of their littermate controls (Fig. 2b). Histomorphometric analysis of Masson trichrome staining showed that the number of osteoblasts per mm of trabecular bone surface (N.Ob/T.Ar) and per mm^2^ of tissue area (Ob.S/Bs) were significantly decreased in *Prx1*^*Cre*^*;Z24*^*fl/fl*^ mice but not in *Osx*^*Cre*^*;Z24*^*fl/fl*^ mice (Fig. 2c–e).

The decreased number of osteoblasts in *Prx1*^*Cre*^*;Z24*^*fl/fl*^ mice prompted us to further examine their self-renewal and apoptosis. We crossed *Prx1*^*Cre*^*;Z24*^*fl/fl*^ mice with mice carrying the *Rosa26*^*Ai9*^ reporter allele, which conditionally expresses the fluorescent tdTomato protein (Ai9) in response to Cre recombination. Cleaved Caspase-3 staining showed an increase in apoptotic cell numbers in the metaphysis and diaphysis (Fig. 2f, g). Staining for the senescence marker SA-β Gal showed increased aging-induced cell senescence in the growth plate and periosteum (Fig. 2h, i). Collectively, these results indicated that premature aging of *Prx1*^*Cre*^-derived cells, but not *Osx*^*Cre*^-derived cells, induced bone loss.

### Two cell subsets were decreased in *Prx1*^*Cre*^ mutant mice

**Fig. 3:**
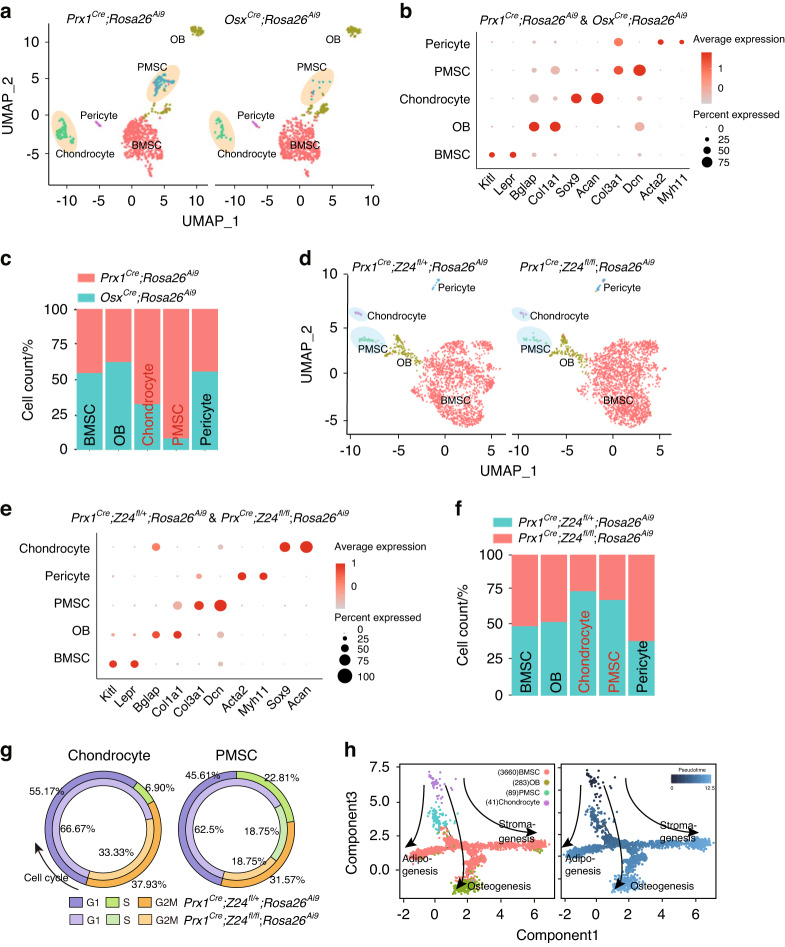
Prx1^Cre+^ Osx^Cre−^ subsets are decreased in *Prx1*^*Cre*^*;Z24*^*fl/fl*^ mice. **a** UMAP plots of 1634 total whole bone Prx1^Cre+^ and Osx^Cre+^ cells from 8-week-old *Prx1*^*Cre*^*;Rosa26*^*Ai9*^ mice and *Osx*^*Cre*^*;Rosa26*^*Ai9*^ mice, colored by cluster assignment and annotated *post hoc*. BMSC: bone marrow stromal cells; OB: osteoblasts; PMSC: periosteum mesenchymal stem cells. **b** Dot plot of gene signatures. **c** Statistical analysis showing the percentages of the five cell clusters (BMSCs, OBs, chondrocytes, PMSCs and pericytes) shown in the panel of *Osx*^*Cre*^*; Rosa26*^*Ai9*^ mice (green) compared to *Prx1*^*Cre*^*; Rosa26*^*Ai9*^ mice (red). **d** UMAP plots of 4119 total whole bone Prx1^Cre+^ cells from three pairs of 16-week-old *Prx1*^*Cre*^*; Z24*^*fl/+*^*; Rosa26*^*Ai9*^ and *Prx1*^*Cre*^*; Z24*^*fl/fl*^*; Rosa26*^*Ai9*^ mice, colored by cluster assignment and annotated *post hoc*. **e** Dot plot showing gene signatures among the 4119 total whole bone cells. **f** Statistical analysis showing the percentages of five cell clusters (BMSCs, OBs, chondrocytes, PMSCs and pericytes) as shown in *Prx1*^*Cre*^*; Z24*^*fl/+*^*; Rosa26*^*Ai9*^ (green) and *Prx1*^*Cre*^*; Z24*^*fl/fl*^*; Rosa26*^*Ai9*^ (red) mice. **g** Cell cycle regression of chondrocyte and PMSC clusters. Inner ring: *Prx1*^*Cre*^*; Z24*^*fl/fl*^*; Rosa26*^*Ai9*^ mice; outer ring: *Prx1*^*Cre*^*; Z24*^*fl/+*^*; Rosa26*^*Ai9*^ mice. **h** Developmental trajectory of the whole bone cells (4073 cells, except for the pericyte cluster) produced by Monocle 2

Given that *Prx1*^*Cre−*^derived cells, but not *Osx*^*Cre−*^derived cells, were found to be responsible for premature aging-induced bone loss, we next used scRNA-seq to compare the cell types between these populations. After quality control and filtering, we obtained CD45^−^Ter119^−^CD31^−^Ai9^+^ single-cell suspensions from 8-week-old *Prx1*^*Cre*^;*Rosa26*^*Ai9*^ (909 cells) and *Osx*^*Cre*^;*Rosa26*^*Ai9*^ (725 cells) mice (including bone marrow and bone fragments) for integrated analysis. Five clusters were mapped to chondrocyte-like cells (chondrocytes, expressing *Sox9* and *Acan*),^18,20^ periosteal mesenchymal stromal cells (PMSCs, expressing *Dcn* and *Col3a1*),^21^ bone marrow stromal cells (BMSCs, expressing *Scf* and *Cxcl12*),^22,23^ pericytes (pericytes, expressing *Acta2* and *Myh11*),^24^ and osteoblasts (OB, expressing *Bglap* and *Col1a1*)^24^ (Fig. 3a, b). Importantly, *Prx1*^*Cre*^*−*derived cells were enriched in chondrocyte-like cells and PMSCs compared to *Osx*^*Cre−*^derived cells (Fig. 3c). We also performed integrated scRNA analyses with 16-week-old *Prx1*^*Cre*^*; Z24*^*fl/+*^*; Rosa26*^*Ai9*^ and *Prx1*^*Cre*^*; Z24*^*fl/fl*^*; Rosa26*^*Ai9*^ mice, which identified the same five clusters (Fig. 3d, e). Strikingly, the percentages of chondrocyte-like cells and PMSCs were decreased in *Prx1*^*Cre*^*;Z24*^*fl/fl*^*; Rosa26*^*Ai9*^ mice (Fig. 3f). Cell cycle analysis showed that chondrocyte-like cells and PMSCs from *Prx1*^*Cre*^*;Z24*^*fl/fl*^*;Rosa26*^*Ai9*^ mice had fewer cells in the S phase (Fig. 3g). Pseudotime analysis (Monocle 2) predicted that chondrocyte-like cells and PMSCs had earlier pseudotimes and had three differentiation trajectories (i.e., osteoblasts, adipocytes and stromal cells) (Fig. 3h, Fig. S3a, b).

**Fig. 4:**
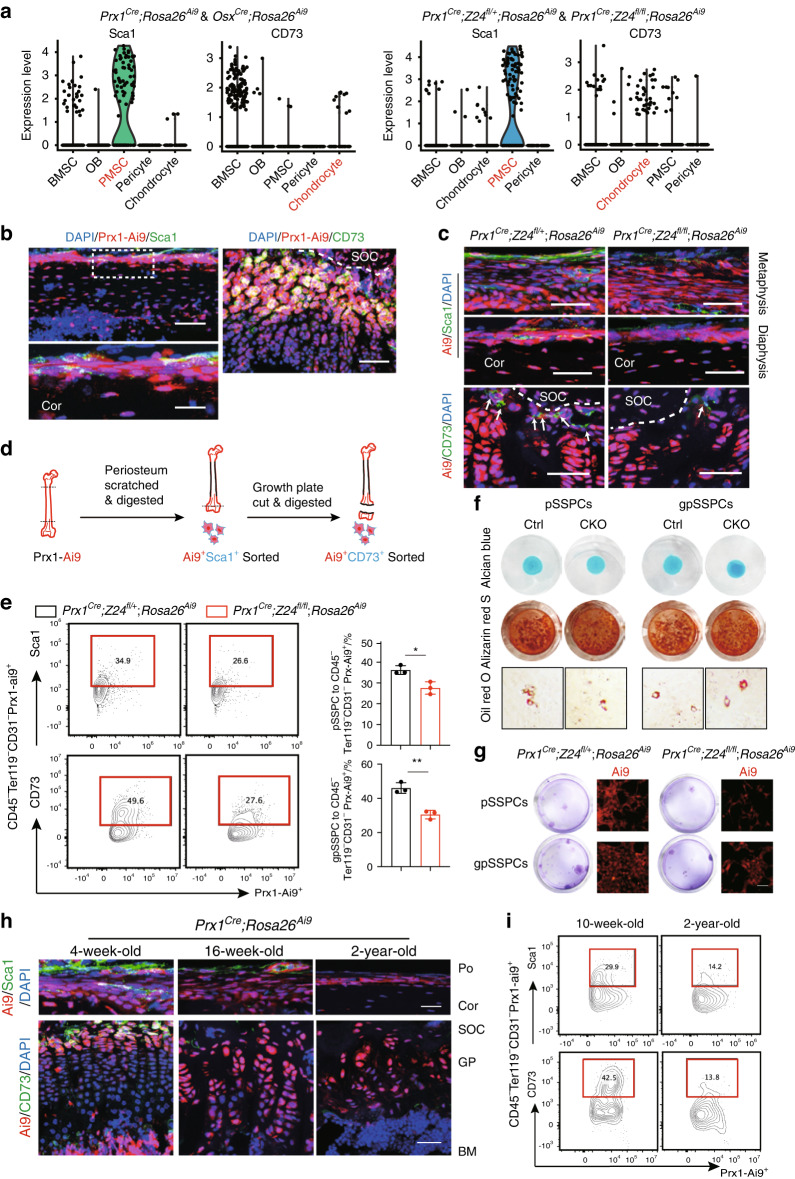
gpSSPC and pSSPC degeneration in both premature and natural aging models. **a** Violin plots showing the expression levels and distribution of the surface markers Sca1 and CD73. **b** Immunofluorescence images of Prx1-Ai9^+^ Sca1^+^ cells and Prx1-Ai9^+^ CD73^+^ cells from 4-week-old *Prx1*^*Cre*^*; Rosa26*^*Ai9*^ mice. Scale bars, Prx1-Ai9^+^ Sca1^+^: 25 μm (top lane), 12 μm (bottom lane); Prx1-Ai9^+^ CD73^+^: 75 μm. **c** Fluorescence images of pSSPCs in the periosteum and gpSSPCs in the growth plate. Scale bars, 50 μm. SOC, secondary ossification center. Cor, cortical bone. **d** Schematic of serial digestion of the Prx1^+^Sca1^+^ (pSSPCs) subset derived from the periosteum and the Prx1^+^CD73^+^ (gpSSPCs) subset derived from the growth plate. **e** Representative FACS analysis of the distribution of the Prx1^+^Sca1^+^ subset derived from the periosteum and the Prx1^+^CD73^+^ subset derived from the growth plate. *n* = 3 per group, data are presented as the mean ± SD. **f** In vitro differentiation of sorted Prx1^+^Sca1^+^ and Prx1^+^CD73^+^ cells from 6-week-old *Prx1*^*Cre*^*; Z24*^*fl/fl*^*; Rosa26*^*Ai9*^ and *Prx1*^*Cre*^*; Z24*^*fl/+*^*; Rosa26*^*Ai9*^ mice into chondrogenic, osteogenic and adipogenic lineages. **g** Colony-formation assay: cells were digested and sorted as shown in (**d**). Left: crystal violet staining; right: enlarged view of the Rosa26^Ai9^ signal. Scale bar, 15 μm. **h** Fluorescence images of pSSPCs in the periosteum (Po) and gpSSPCs in the growth plate (GP) at 4 weeks, 16 weeks and 2 years in *Prx1*^*Cre*^*; Rosa26*^*Ai9*^ mice. Scale bars, 50 μm (top lane) and 40 μm (bottom lane). BM, bone marrow. **i** FACS analysis of the distribution of gpSSPCs and pSSPCs in 10-week-old and 2-year-old *Prx1*^*Cre*^*;Rosa26*^*Ai9*^ mice

Next, we selected the top-ranked membrane proteins Sca1 and CD73 from sequencing data and used them as marker genes of PMSCs and chondrocyte-like cells, respectively (Fig. 4a). Consistent with recent studies, immunostaining data confirmed the expression of Sca1 and CD73 in Prx1-Ai9-expressing periosteal cells and growth plate resting zone cells, respectively (Fig. 4b).^5,25–28^ These cells are named periosteal skeletal stem/progenitor cells (pSSPCs, *Prx1-Ai9*^+^ Sca1^+^) and growth plate skeletal stem/progenitor cells (gpSSPCs, *Prx1-Ai9*^*+*^ CD73^+^) hereafter. Immunostaining analysis confirmed that pSSPCs and gpSSPCs were significantly decreased in *Prx1*^*Cre*^*;Z24*^*fl/fl*^*;Rosa26*^*Ai9*^ mice (Fig. 4c). We digested the long bones and flow-sorted pSSPCs and gpSSPCs from the periosteum and growth plate, the frequency of these cells was decreased in *Prx1*^*Cre*^*;Z24*^*fl/fl*^*;Rosa26*^*Ai9*^ mice, which was consistent with immunostaining data (Fig. 4d, e). Trilineage differentiation analyses showed that these cells have similar capacities to differentiate into chondrocytes, osteoblasts and adipocytes in vitro (Fig. 4f). However, both pSSPCs and gpSSPCs from *Prx1*^*Cre*^*;Z24*^*fl/fl*^*;Rosa26*^*Ai9*^ mice exhibited lower colony-formation abilities and weaker self-renewal abilities (Fig. 4g). Furthermore, immunostaining revealed a decrease in pSSPC and gpSSPC numbers during aging (Fig. 4h). Flow cytometry analysis also confirmed a decrease in both pSSPC and gpSSPC numbers in 2-year-old aged mice compared with 10-week-old young mice (Fig. 4i). Taken together, these results demonstrated the decrease in the pSSPC and gpSSPC populations in both premature and physiological aging mouse models, highlighting their critical roles in maintaining adult bone mass.

### Premature aging of gpSSPCs leads to trabecular bone loss

**Fig. 5:**
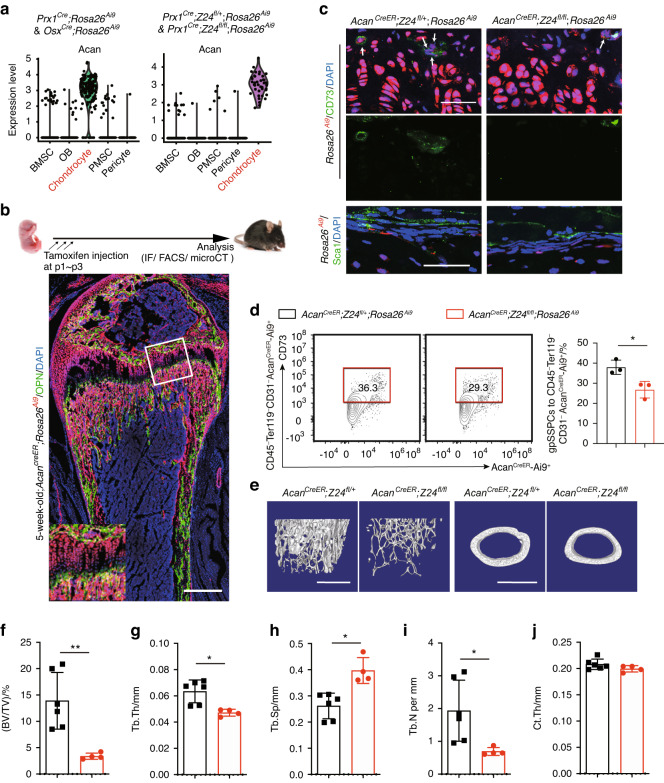
*Acan*^*CreER*^*;Z24*^*fl/fl*^ mice exhibit trabecular bone loss. **a** Violin plots showing the expression levels and distribution of *Acan*. **b** Fluorescence images of OPN in femur sections from *Acan*^*CreER*^*; Rosa26*^*Ai9*^ mice that were injected with tamoxifen at p1-p3. Mice were analyzed after 5 weeks. Scale bar, 0.5 mm. **c** Fluorescence images of CD73 in the growth plate and Sca1 in the periosteum of 16-week-old *Acan*^*CreER*^*; Z24*^*fl/fl*^*; Rosa26*^*Ai9*^ mice and *Acan*^*CreER*^*; Z24*^*fl/+*^*; Rosa26*^*Ai9*^ mice. Scale bars, 50 μm. **d** FACS analysis of the distribution of Acan^+^CD73^+^ cells from growth plates of 8-week-old *Acan*^*CreER*^*; Z24*^*fl/+*^*; Rosa26*^*Ai9*^ and *Acan*^*CreER*^*; Z24*^*fl/fl*^*; Rosa26*^*Ai9*^ mice that were injected with tamoxifen at p1-p3. *n* = 3 per group. Data are presented as the mean ± SD. **e** MicroCT images showing femurs of 16-week-old *Acan*^*CreER*^*; Z24*^*fl/fl*^ mice and their littermate controls. Scale bars, 1 mm. Quantitative analyses of trabecular bone volume/total volume (BV/TV) (**f**), trabecular thickness (Tb.Th) (**g**), trabecular spacing (Tb.Sp) (**h**), trabecular number (Tb.N) (**i**) and cortical thickness (Ct.Th) (**j**) in the femur metaphysis of 16-week-old *Acan*^*CreER*^*; Z24*^*fl/fl*^ mice (*n* = 4) and their littermate controls (*n* = 6). The statistical significance of differences was assessed using two-tailed Student’s paired *t* test (*P < 0.05, **P < 0.01, ***P < 0.001 and ****P < 0.000 1)

To verify whether Z24 in gpSSPCs plays a role in vivo, we proposed to generate gpSSPC-specific conditional Z24-knockout mice. Chondrocytes express high levels of aggrecan (*Acan*), type II collagen (*Col2*) and *Sox9* and have been shown to progressively contribute to lifelong skeletal lineages.^18^ In our scRNA-seq results, gpSSPCs exhibited chondrocyte characteristics, raising the possibility that *Z24* deficiency in chondrocytes is sufficient to induce bone loss (Fig. 5a). Acan is also a highly ranked marker of chondrocyte clusters. Therefore, we crossed *Acan*^*CreER*^ mice with *Z24*^*fl/fl*^*;Rosa26*^*Ai9*^ mice to directly test the cellular role of gpSSPCs temporally. Consistent with the data we obtained, most osteoblasts in trabecular bones could be labeled by *Acan*^*CreER*^*;Rosa26*^*Ai9*^ when tamoxifen was administered at p1-p3 (Fig. 5b). Immunostaining showed a significant decrease in *Acan-Ai9*^+^CD73^+^ cell numbers in *Acan*^*CreER*^*;Z24*^*fl/fl*^*;Rosa26*^*Ai9*^ mice compared to control mice, which was consistent with flow cytometry analysis (Fig. 5c, d). MicroCT analysis of *Acan*^*CreER*^*;Z24*^*fl/fl*^ mice showed significantly decreased trabecular bone volume fraction, trabecular thickness, and trabecular number and significantly increased trabecular spacing compared to littermate control mice (Fig. 5e–i). Since *Acan*^*CreER*^ cannot label Sca1^+^ pSSPCs, there was no significant difference in cortical thickness (Ct.Th) (Fig. 5c, j). The data further suggested that the decline in *Prx1-Ai9*^*+*^ CD73^+^ gpSSPC numbers is responsible for the impairment of aged trabecular bone.

### Premature aging of SSPCs enhances the apoptotic response and decreases the production of extracellular matrix associated with mechanotransduction

**Fig. 6:**
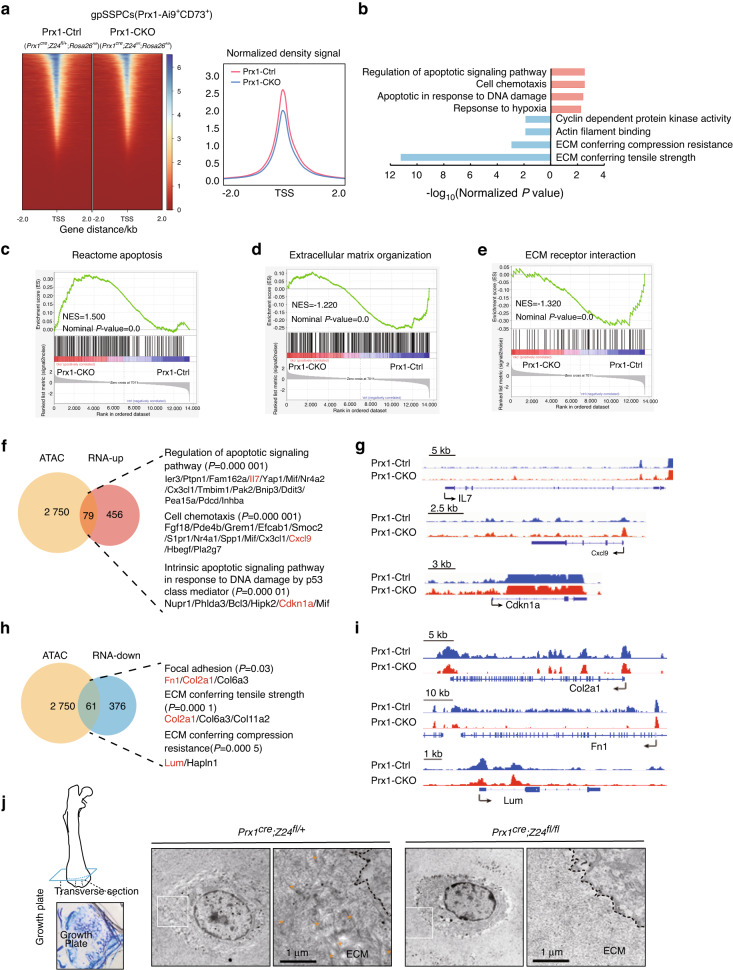
Apoptosis pathway and extracellular matrix degradation in prematurely aged gpSSPCs. **a** Normalized ATAC-seq signal intensity around the transcription start site (TSS). *n* = 3 per group. Right histograms show genome-wide chromatin accessibility at the TSS. **b** GO terms enriched for significantly different genes identified by RNA-seq. Selected GO terms with significant *P* values are shown. GSEA of the expression of Reactome apoptosis signatures (**c**), extracellular matrix organization (**d**) and ECM receptor interaction (**e**) in gpSSPCs. *n* = 3 per group. Venn diagram (**f**) showing the overlap of genes with open chromatin in close proximity and transcripts and selected ATAC-seq signal tracks (**g**) that are differentially upregulated in gpSSPCs. The number of genes near peaks that are accessible and significantly differentially expressed genes is shown. Venn diagram (**h**) showing the overlap of genes with open chromatin in close proximity and transcripts and selected ATAC-seq signal tracks (**i**) that are differentially downregulated in gpSSPCs. The number of genes near peaks that are accessible and significantly differentially expressed genes is shown. **j** TEM images of the epiphyseal plate. Left: TEM methodology. Consecutive thin transversal sections of the epiphyseal plate analyzed by toluidine blue. Scale bars, 1 μm

To further explore the potential molecular mechanisms underlying gpSSPC and pSSPC senescence, we obtained gpSSPCs by flow cytometry and performed an assay for targeting accessible chromatin with high-throughput sequencing (ATAC-seq) and RNA sequencing (RNA-seq). The accessibility of chromatin regions flanking the transcriptional start sites (TSSs) was significantly lower, suggesting decreased transcriptional activity (Fig. 6a, Fig. S4a). Gene Ontology (GO)/KEGG analysis revealed an enrichment of upregulated genes related to apoptotic signaling, chronic inflammation and p53-mediated DNA damage response (Fig. 6b, Fig. S4b). Gene set enrichment analysis (GSEA) revealed that *Z24*-deficient gpSSPCs and pSSPCs were enriched in genes related to apoptosis (Fig. 6c, Fig. S4c). By integrating analysis of the ATAC-seq and RNA-seq data, we found 79 and 416 upregulated genes with differentially opened chromatin regions, respectively (Fig. 6f, Fig. S4f). As expected, the chromatin regions of upregulated genes (gpSSPCs: *IL7*, *Cxcl9* and *Cdkn1a*; pSSPCs: *Gbp6*, *Snai2* and *Cdkn1a*) displayed higher accessibility (Fig. 6g, Fig. S4g).

GO/KEGG pathway analyses revealed that downregulated genes were related to ECM-associated proteins (Fig. 6b, Fig. S4b). GSEA showed a downregulation of genes related to ECM organization and ECM receptor interaction in *Z24*-deficient gpSSPCs, downregulation of invasion inhibited by ascites and upregulation of the uterine fibroid signature in *Z24*-deficient pSSPCs (Fig. 6d, e, Fig. S4d, e). Integrated analysis revealed 61 and 551 downregulated genes with differentially opened chromatin regions in *Z24*-deficient gpSSPCs and pSSPCs, respectively (Fig. 6h, Fig. S4h). As expected, the chromatin regions of downregulated genes (gpSSPCs: *Col2a1*, *Fn1* and *Lum*; pSSPCs: *Col5a1*, *Itga2*, *Itga11* and *Aspn*) exhibited lower accessibility in *Z24*-deficient cells (Fig. 6i, Fig. S4i). Furthermore, transmission electron microscopy (TEM) images showed that *Z24*-deficient gpSSPCs and pSSPCs were embedded in disordered ECM structures (Fig. 6j, Fig. S4j). Given that ECM provides physical scaffolds and essential biomechanical cues for cell migration, differentiation, and homeostasis, changes in ECM structures may promote the degeneration of *Z24*-deficient gpSSPCs and pSSPCs.

Furthermore, we aimed to determine whether there is any difference between differentially expressed genes (DEGs) in gpSSPCs and pSSPCs, and we found 28 genes (such as *Cdkn1a*, *Bcl3*, and *Cxcl9*) with increased expression and 23 genes (such as *Nrde2*, *Tnn*, and *Lrp4*) with decreased expression in both pSSPCs and gpSSPCs; these results suggested that the deletion of *Z24* played a similar role in these two SSPC populations. Moreover, transcriptome sequencing analysis revealed that the expression of additional genes was changed only in gpSSPCs (upregulated: *Serpina1a/b/c/d/e*, *Apoe*, and *Wif1*; downregulated: *Anxa9*, *Nadsun1*, and *Cavin4*) or pSSPCs (upregulated: *Slpi*, *Prg4*, and *Rgcc*; downregulated: *Inmt*, *Myoc*, and *Slurp1*); these results suggested the specificity of the effects of Z24 on different cell subsets (Table S3).

### Mechanical stimulation promotes SSPC numbers and alleviates bone loss

Based on the bioinformatics analysis that suggested a link between aging due to Z24 deletion and biomechanics, we next verified whether biomechanical stimulation could reverse this phenotype in premature aging model mice. First, 8-week-old C57BL/6J mice were subjected to medium-intensity treadmill running for 5 weeks to investigate the changes in SSPCs (Fig. S5a, Table S1). MicroCT analysis confirmed that treadmill exercise increased bone density, especially trabecular bone mass (Fig. S5b–g). Histological analysis was conducted on sections of femurs to examine SSPC responses to mechanical signals. Immunostaining of the growth plate showed a significant increase in the number of CD73^+^ cells (gpSSPCs) after 5 weeks of treadmill running (Fig. S5h).

To better understand the relationship between prematurely aged gpSSPCs and bones subjected to exercise, we performed RNA sequencing of bones after 5 week of exercise and compared their differentially expressed genes. We found that 64 genes were overrepresented in prematurely aged gpSSPCs and decreased in bones subjected to exercise, and 22 genes showed the opposite trend, especially *p21*/*Cdkn1a* (Fig. S6a–d). The apoptotic signaling pathway in response to DNA damage was among the GO terms enriched for these 64 genes (Fig. S6e), indicating that the apoptosis induced by the Prelamin A-induced decreased mechanosensation might be reversed by physical exercise.

**Fig. 7:**
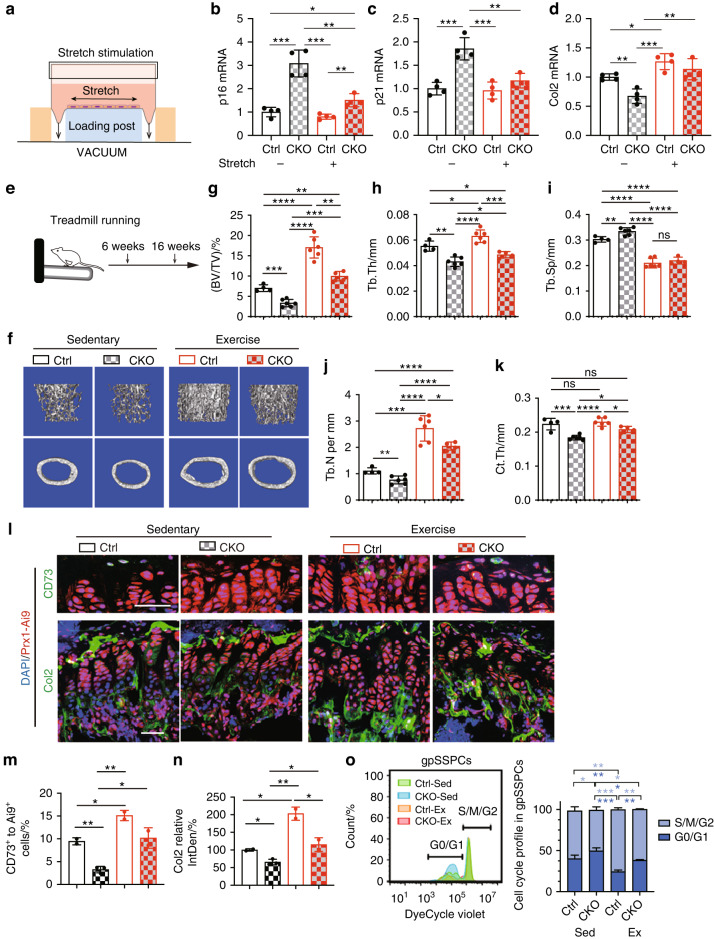
Mechanical stimuli promote SSPC numbers. **a** Schematic showing the stretch stimulation system. **b–d** Gene expression of *p16/Cdkn2a*, *p21/Cdkn1a* and *Col2a1* in bone marrow stromal cells of *Prx1*^*Cre*^*; Z24*^*fl/fl*^ (CKO) and *Z24*^*fl/fl*^ (Ctrl) mice stimulated at 3% intensity and 0.5 Hz for 4 h with the Flexcell-5000C^TM^ Tension System. **e** Schematic showing the treadmill experimental strategy. **f** Representative microCT images of femurs. Quantitative analyses of trabecular bone volume/total volume (BV/TV) (**g**), trabecular thickness (Tb.Th) (**h**), trabecular spacing (Tb.Sp) (**i**), trabecular number (Tb.N) (**j**) and cortical thickness (Ct.Th) (**k**) in the femurs. The statistical significance of differences was assessed using two-tailed Student’s paired *t* test (*P < 0.05, **P < 0.01, ***P < 0.001 and ****P < 0.000 1). **l** Fluorescence images of CD73 and Col2 in the growth plate from 16-week-old WT and CKO mice that exercised for 10 weeks. Sedentary littermates were used as controls. Scale bar, 50 μm. **m** Proportion of CD73^+^ cells to *Prx1*^*Cre*^*; Rosa26*^*Ai9*^ cells in tissue sections (*n* = 2). **n** Quantification of the fluorescence intensity of Col2 in tissue sections using ImageJ software, IntDen: integrated density (*n* = 2). **o** Flow cytometry of the gpSSPC cell cycle after running (*n* = 3). The statistical significance of differences was assessed using two-tailed Student’s paired *t* test (*P < 0.05, **P < 0.01, ***P < 0.001 and ****P < 0.000 1)

Then, we used stretch stimulation to examine the effects of mechanical stimuli on prematurely aged cells (Fig. 7a). According to senescence marker (*p16*/*Cdkn2a* and *p21*/*Cdkn1a*) expression, cell senescence in *Z24*-deficient bone marrow stromal cells was attenuated by stretching, as shown by the transcriptional level (Fig. 7b, c). Furthermore, extracellular matrix proteins, such as *Col2a1*, were enhanced after stretching (Fig. 7d). We collected RNA from bone tissues of exercised mice, and the results were consistent with our in vitro results (Fig. S7a–c). Staining for the apoptosis marker cleaved Caspase-3 showed a sharp decrease in apoptotic cell numbers in the *Z24*-deficient growth plate after running compared to those in the *Z24*-deficient growth plate of sedentary mice (Fig. S7d). These results suggested that mechanical stimuli could be used to reverse the apoptosis response and cell senescence in prematurely aged cells. Therefore, we subjected *Z24*-deficient mice to treadmill running for 10 weeks (Fig. 7e, Table S2).

MicroCT analysis showed an increase in bone mass in CKO mice after exercise, as bone volume/tissue volume and trabecular numbers were significantly increased after 10 weeks of running compared with sedentary WT mice (Fig. 7f, g, i, j). Compared with sedentary CKO mice, trabecular thickness and cortical thickness were increased after running (Fig. 7h, k). Consistent with 5 weeks treadmill running, immunostaining of the growth plate showed a significant increase in the number of CD73^+^ cells and extracellular matrix production, such as *Col2a1*, in *Prx1*^*Cre*^*;Z24*^*fl/fl*^ mice (Fig. 7l–n). Cell cycle analysis of gpSSPCs (*Prx1-Ai9*^*+*^ CD73^+^) by flow cytometry showed that G0/G1 phase arrest in sedentary CKO mice was reversed by exercise (Fig. 7o). These data suggested that running increased SSPC numbers, reversed cell apoptosis/cell cycle arrest and increased ECM production in prematurely aged mice.

**Fig. 8:**
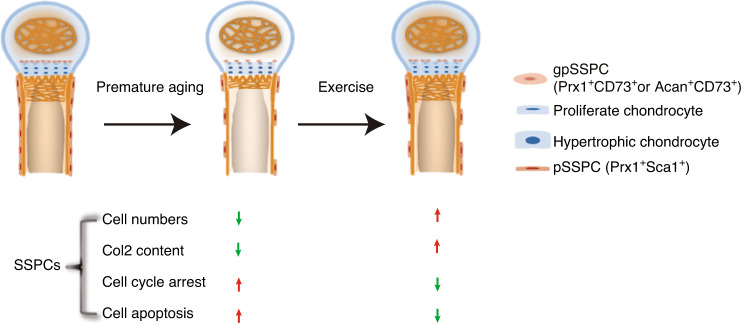
Schematic representation of key finding. Schematic showing key findings. Mechanical stimuli increase SSPC numbers and ECM (Col2) content and prevent cell cycle arrest and cell apoptosis in prematurely aged mice

Taken together, our work is the first to establish animal models of premature aging specifically in skeletal stem cells. Combined with single-cell sequencing, we demonstrated the effect of reduced gpSSPC and pSSPC numbers on bone loss in the premature aging model. The effects of cellular senescence on apoptosis, proliferation, extracellular matrix production and biomechanical stimulation were elucidated in depth using ATAC sequencing and RNA sequencing. We also demonstrated that exercise could increase the number of SSPCs to alleviate bone loss (Fig. 8). This will provide new insights into the understanding of osteoporosis and provide evidence for the development of stem cell therapy for osteoporosis and training programs for osteoporosis relief.

## Discussion

Taking advantage of the *Cre*/*LoxP* system and mouse models of premature aging, we generated a model of conditional premature aging specifically in skeletal cells. By scRNA-seq, we showed that the numbers of pSSPCs and gpSSPCs, which were enriched among *Prx1*-*Cre*-derived cells compared to *Osx*-*Cre*-derived cells, were decreased in premature and naturally aging mice, supporting the hypothesis that the decline in these two populations is responsible for age-related bone loss. Considering a remodeling unit between osteoblasts and osteoclasts, changes in the number of osteoblasts could affect osteoclastogenesis. However, we found that osteoclast function was indistinguishable at 8 weeks of age; however, by 16 weeks, osteoclast function was slightly reduced in *Prx1*^*Cre*^*;Z24*^*fl/fl*^ mice when their bone mass was significantly decreased (Fig. S2h, i). Later, the function of osteoclasts began to be gradually suppressed by the effects of osteoblasts. In addition, we found that cell chemotaxis and response to INFγ pathways were upregulated (Fig. 6f and Fig. S4f) according to the transcriptome analysis, and these effects also enhanced osteoclast function. These positive and negative effects on osteoclasts may have resulted in indistinguishable changes in function in the *Prx1*^*Cre*^*;Z24*^*fl/fl*^ mouse model.

Recently, Ambrosi et al. identified osteochondral and perivascular skeletal stem cells (ocSSCs and pvSSCs) according to their differentiation potential and proved that aged SSCs decreased bone and cartilage formation and increased stroma production.^29,30^ According to cell regional specialization, our study further investigated the cellular and molecular mechanisms of SSPCs using a genetic conditional premature mouse model. In terms of’the top differentially expressed genes in ocSSCs and pvSSCs, gpSSPCs (*Prx1-Ai9*^*+*^ CD73^+^) are more similar to ocSSCs, while pSSPCs (*Prx1-Ai9*^*+*^ Sca1^+^) seem to be more similar to pvSSCs (CD45^−^CD31^−^Sca1^+^CD24^+^), which have multilineage potential to give rise to adipogenic progenitor cells and osteochondrogenic progenitor cells (Fig. S3c).^30,31^ In addition, Prx1^+^Sca1^+^ cells have been considered a more primitive osteogenic population.^25,32^ In accordance with Cabahug-Zuckerman et al., our IF and flow cytometric analyses revealed that pSSPC numbers decreased in naturally and prematurely aged mice, which suggested that pvSSCs in the periosteum differed from pvSSCs in bone marrow after aging.^25,30^ We aimed to identify membrane proteins for fluorescence-activated cell sorting (FACS) to establish SSPC characteristics. In our scRNA sequencing profile, Sca1 and CD73 were the top-ranked membrane protein markers of PMSC and chondrocyte clusters, respectively. In addition, we used the genetic model *Acan*^*CreER*^, which is also a highly ranked marker of chondrocyte clusters, to assess CD73^+^ chondrocyte-like cell (gpSSPC) function in vivo.

Integrated analysis showed disordered ECM production in premature aged gpSSPCs and pSSPCs, indicating abnormal mechanosensitivity in these cells. Lineage specification and cell differentiation of SSPCs^33^ and mesenchymal stem cells (MSCs)^34^ have been shown to be extremely sensitive to extracellular matrix elasticity and mechanical stimuli. Aging influences the ability of cells to sense changes in the extracellular matrix and to transduce these changes into biochemical signals (mechanotransduction).^35^ In addition to the extracellular matrix and the cytoskeleton, the nucleus is considered a key mechanosensor^36^ and can directly influence chromatin organization, epigenetic modifications and gene expression in response to the microenvironment of a cell. Z24 deficiency^15,16^ has been associated with human diseases that are characterized by severe defects in nuclear stability, cytoskeletal dynamics and nucleocytoskeletal force transmission. Here, the conditional deficiency of Z24 in our mouse model changed nuclear mechanical stimuli, allowing us to explore its mechanism in SSPCs.

HGPS is a human progeroid syndrome and is considered the ‘Rosetta Stone’ for studying the mechanisms of aging.^37^ It is worth noting that HGPS exhibits minimal defects in the nervous system and immune system, and our current model may only partially represent the multifactorial process of normal aging.^38^ However, our conditional premature aging model could be a useful tool to study interorgan homeostasis, for example, how aged skeletons affect other tissues and organs.

## Materials and methods

### Mice

*Zmpste24-KO* first mice were purchased from the CAM-SU Genomic Resource Center (Clone No: EPD0705_3_A07). *Prx1-Cre* (stock no: 005584) mice, *Osx-Cre* (stock no: 006361) mice and *Acan-CreER* (stock no: 019148) mice were obtained from the Jackson Laboratory and were maintained in the laboratory for more than 10 generations. Ai9 reporter mice were provided by Zilong Qiu (Chinese Academy of Sciences, Shanghai, China). *Acan*^*CreER*^ mice are commonly used mice in the laboratory and have been maintained in the laboratory for more than 10 generations. We crossed *Acn*^*CreER*^ mice with *Z24*^*fl/fl*^ mice to obtain F1 generation mice (*Acan*^*CreER*^*; Z24*^*fl/+*^) and crossed the F1 generation mice with *Z24*^*fl/fl*^ mice to obtain target genotype mice. All the mice were on a C57BL/6 background, and sex-matched littermate controls were used for all the analyses. The genotyping primers that were used are shown in Table 1.

**Table 1:** Genotyping primers

Gene name	Sequence
Zmpste24-KO first-F	TCATCACAGACAATCATACAGGCAAGG
Zmpste24-KO first-WT-R	GAGATAAAGCAACAAACGCCACA
Zmpste24-KO first-KO-R	TGGGACCACCTCATCAGAAGCAG
Zmpste24^fl/fl^-F	GTTCTTGTCACCATTTATGCTGAC
Zmpste24^fl/fl^-R	CTTCAACAACATACACCTTAGTCA
Ai9-1	AAGGGAGCTGCAGTGGAGTA
Ai9-2	CCGAAAATCTGTGGGAAGTC
Ai9-3	GGCATTAAAGCAGCGTATCC
Ai9-4	CTGTTCCTGTACGGCATGG
Cre^ER^/Cre-F	CGATGCAACGAGTGATGAGG
Cre^ER^/Cre-R	CGCATAACCAGTGAAACAGC

### MicroCT analysis

Mouse femurs were skinned and fixed in 70% ethanol. Scanning was performed using a SkyScan1276 (Bruker, Kartuizersweg, Belgium) in vivo microCT at a 9 μm resolution for quantitative analysis. The region from −100 to −300 slides below the growth plate was analyzed to determine trabecular number, trabecular thickness, trabecular spacing, and bone volume per tissue volume with CTAn. The region from −480 to −536 slides below the growth plate was analyzed to determine cortical thickness. Three-dimensional reconstructions were created by stacking the two-dimensional images from the indicated regions with CTVox52 software.

### Treadmill exercise

Medium-intensity treadmill exercise was performed as previously described.^39^ Mice were placed on the SA101S treadmill (SANS Biotechnology, China). Electric shock was on during the whole experiment. Mice of different ages were subjected to different durations of exercise. Please see Tables S1 and S2.

### Tamoxifen treatment

Tamoxifen was dissolved in corn oil at a concentration of 20 mg·mL^−1^ by shaking overnight at 37 °C (tube was protected from light with tin foil). For the neonatal induction of Cre^ER^ activity, mice were intraperitoneally injected with 4-OH tamoxifen (50 μg, Sigma) at on days 1–3 using an insulin syringe (BD).

### Real-time RT‒PCR analysis

Total RNA was prepared using TRIzol (Sigma, T9424) and reverse transcribed into cDNA with the PrimeScript RT Reagent Kit (Takara, PR037A). Real-time reverse transcriptase RT-PCR was performed with the Bio-Rad CFX96 system. The qPCR primers used are shown in Table 2.

**Table 2:** qPCR primers

Gene name	Sequence
Zmpste24-F	TTTCTCTGGTTCTTGTCAC
Zmpste24-R	AACGCTTAGATCCTTCAAC
Hprt-F	GTTAAGCAGTACAGCCCCAAA
Hprt-R	AGGGCATATCCAACAACAAACTT
p16-F	CTAGAGAGGATCTTGAGAAGAGGGC
p16-R	TAGTTGAGCAGAAGAGCTGCTACGT
p21-F	TCAGAGTCTAGGGGAATTGGA
p21-R	AATCACGGCGCAACTGCT
Col2a1-F	CGGTCCTACGGTGTCAGG
Col2a1-R	GCAGAGGACATTCCCAGTGT

### Cell stretch experiment

Bone marrow stromal cells (BMSCs) were seeded at 2 × 10^5^ cells per well with α-MEM (supplemented with 10% FBS and 1% penicillin‒streptomycin) in six-well collagen-coated BioFlex Plates (Flexcell). The Flexcell-5000C^TM^ Tension System (Flexcell Corporation) was used to provide regulated strain. BMSCs were cultured in a humidified atmosphere of 5% CO_2_ at 37 °C and stimulated with mechanical strain at 80% confluence at 3% intensity, 0.5 Hz for 4 h. As a control, cells were cultured in the same BioFlex Plates with the same conditions but without being subjected to stretch force.

### Western blotting

Tissues were disrupted using a homogenizer and lysed with NP40 lysis buffer supplemented with protease inhibitor and phosphatase inhibitor cocktail (Selleck). Protein samples were separated by SDS‒PAGE and transferred to nitrocellulose filter membranes (Millipore). The membranes were blocked with 5% nonfat milk (in TBST) and incubated with primary antibodies against Lamin A/C (Abcam, ab108922, 1:500), P16 (Cell Signaling Technology, 1:2 000) and Tubulin (Santa Cruz Biotechnology Inc., 1:1 000) at 4 °C overnight. The proteins were visualized using horseradish peroxidase-conjugated (HRP-conjugated) secondary antibodies and chemiluminescent HRP substrate (Millipore).

### Histology analysis

#### Skeletal whole-mount staining

Mice were eviscerated, and the skin was removed. The resulting samples were incubated in acetone for 48 h after overnight fixation in 95% ethanol. Skeletons were then stained in Alcian blue and Alizarin red solution as previously described. Specimens were stored in 1% KOH until the tissue had completely cleared.

#### Calcein double staining

Mice were intraperitoneally injected with 20 mg·kg^−1^ Calcein (1 mg·mL^−1^ in 2% NaHCO_3_ solution) on day 0 and day 7 separately. On day 12, the mice were sacrificed, and the bones were fixed in 4% paraformaldehyde (PFA), dehydrated and embedded with the EMbed 812 Kit (Electron Microscopy Sciences). The samples were sectioned at 5 μm with a hard tissue cutter. Consecutive sections were stained with silver nitrate to measure the bone morphology. The histomorphometric analysis was carried out semiautomatically with an OsteoMeasure image analyzer (OsteoMetrics) according to recommended guidelines.

#### Safranin O (SO) and TRAP staining

Freshly dissected bones were fixed in 4% PFA for 48 h and incubated in 15% EDTA (pH 7.8) for decalcification. Specimens were then embedded in paraffin and sectioned at 7 μm using a Leica RM2235 microtome. Next, the paraffin sections were dewaxed, hydrated and stained with SO. The tissue sections were subjected to TRAP staining according to the manufacturer’s instructions (Sigma, 387A-1KT). Images were captured using an upright microscope (Olympus BX53).

#### Immunostaining

Freshly dissected bones were fixed in 4% PFA for 48 h, incubated in 15% EDTA (pH 7.8) for decalcification, and dehydrated in 30% sucrose for 48 h. Then, the specimens were embedded in OCT medium (Leica, 14020108926) and sectioned at 14 μm using a Leica CM3050S cryostat. Frozen sections were air-dried and rehydrated with PBS. Antigen retrieval was performed either with pepsin at 37 °C for 30 min or with citrate buffer solution (pH 6.0) at 95 °C for 15 min according to the specific instructions for the primary antibody. After blocking and permeabilization with 10% horse serum and 0.2% Triton-X 100 in PBS for 1 h at room temperature, the sections were incubated with primary antibodies overnight at 4 °C. The primary antibodies included rabbit anti-LacZ (Abcam, Ab9361, 1:200), goat anti-Opn (R&D, AF808, 1:500), rabbit anti-cleaved Caspase-3 (Cell Signaling Technology, 9661, 1:200), mouse anti-Col2 (Abcam, ab185430, 1:500), rat anti-Sca1 (Biolegend, 108120, 1:500) and rat anti-CD73 (Biolegend, 127217, 1:500). Fluorescence-labeled secondary antibodies included donkey anti-rabbit Alexa Fluor 647 (Molecular Probes, A31573, 1:1 000), donkey anti-goat Alexa Fluor 647 (Molecular Probes, A21447, 1:1 000) and goat anti-rat Alexa Fluor 647 (Molecular Probes, A21247, 1:1 000). DAPI (Sigma, D8417) was used for counterstaining. The cell senescence detection kit SPiDER-β Gal was purchased from Dojindo (SG03). Slides were mounted with anti-fade fluorescence mounting medium (Dako, S3023), and images were acquired with a confocal microscope (SP8 WLL, Leica).

#### Transmission electron microscopy (TEM)

To prepare samples for TEM, organoids or islets were fixed in 1% glutaraldehyde in 0.1 mol·L^−1^ sodium cacodylate buffer (pH 7.4) at 4 °C overnight and postfixed in 2% aqueous osmium tetraoxide at 4 °C for 1.5 h. The samples were then dehydrated in increasing concentrations of ethanol (30%–100%) and propylene oxide, embedded in Epon 812 and incubated for 48 h at 60 °C. Ultrathin sections (50 nm) were collected onto 200 mesh copper grids and stained with uranyl acetate (10 min) and lead citrate (5 min). Images were captured with an FEI Tecnai G2 Spirit transmission electron microscope.

### Enzymatic dissociation and flow cytometry

#### Bone and bone marrow cells (for scRNA-seq)

To isolate bone and bone marrow cells, marrow plugs were flushed with a 1 mL syringe using staining buffer (1x HBSS + 2% FBS) and digested 3 times for 5 min each. The remaining bone fragments were crushed and digested following the same methods as the marrow plugs. The digestion buffer contained 2 mg·mL^−1^ Collagenase II (Sigma, C6885), 2 mg·mL^−1^ Dispase II (Roche), 200 U·mL^−1^ DNase I, and 10 mg·mL^−1^ Kolliphor P 188 (Sigma, 15759) in 1× HBSS with Mg^2+^/Ca^2+^. The bone and marrow fractions were pooled and filtered through 70 μm cell strainers, washed twice with ice-cold staining buffer and centrifugation at 600 × *g* for 5 min, and incubated for 30 min on ice with the following antibodies: PerCP/Cy5.5 anti-CD31 (BioLegend, 102420), PerCP/Cy5.5 anti-CD45 (BioLegend, 103132), and PerCP/Cy5.5 anti-mouse Ter-119 (BioLegend, 116228). The cells were then sorted on an MA900 flow cytometer (Sony) with a 100 μm nozzle.

#### Periosteal and growth plate cells (for bulk RNA-seq, ATAC-seq and cell culture)

To isolate periosteal cells,^40^ femurs and tibiae were placed in ice-cold PBS after the overlying skin and muscle were carefully removed. The periosteum was gently scratched using a scalpel and forceps and then incubated with prewarmed digestion buffer at 37 °C for 30 min on a shaker. The dissociated periosteal cells were washed twice by centrifugation at 600 × *g* for 5 min with ice-cold staining buffer.

To isolate growth plate cells,^28^ dissected growth plates were minced using a scalpel and incubated with 0.15% collagenase (Sigma, C6885) at 37 °C for 90 min on a shaking incubator. The cells were pelleted and resuspended in ice-cold staining buffer.

The cells were stained with PerCP/Cy5.5 anti-CD31 (BioLegend, 102420), PerCP/Cy5.5 anti-CD45 (BioLegend, 103132), PerCP/Cy5.5 anti-mouse Ter-119 (BioLegend, 116228), BV421 anti-mouse CD73 (BioLegend, 127217) or Pacific blue anti-Sca1 (BioLegend, 108120) for 30 min and sorted on an MA900 flow cytometer (Sony) with a 100 μm nozzle.

### Cell cycle analysis

Cell cycle analysis was performed using DyeCycleTM Violet Stain (Thermo Fisher). Growth plate skeletal stem/progenitor cells (gpSSPCs) were first stained with antibodies against their surface markers (CD31^−^CD45^−^Ter119^−^CD73^+^) followed by permeabilization and fixation using BD Cytofix/Cytoperm (BD Biosciences) and intracellular labeling.

### CFU-F assays and cellular multipotent differentiation

For colony-formation assays, 1 000 sorted Prx1^+^CD73^+^ and Prx1^+^Sca1^+^ cells from the growth plate and periosteum of 8-week-old *Prx1*^*Cre*^*;Z24*^*fl/fl*^*;Rosa26*^*Ai9*^ and *Prx1*^*Cre*^*;Rosa26*^*Ai9*^ mice were cultured for 14 days at 37 °C in 5% CO_2_. CFU-F-derived colonies were observed using a fluorescence microscope (Olympus, IX73). Then, the cells were fixed in 4% PFA and stained with crystal violet. Images were captured using an upright microscope (Olympus BX53).

For osteoblast differentiation, cells were cultured in α-MEM supplemented with 10% FBS, 1% penicillin/streptomycin, 50 μg·mL^−1^ l-ascorbic acid (Sigma, A5960), and 1.08 mg·mL^−1^ β-glycerophosphate disodium salt hydrate (Sigma, G9422). The medium was changed every 3 days. Cells were analyzed using a luminometer (Envision) at A405 to determine ALP activity. The final Alp activity was normalized as A405/alamarBlue. Bone nodule formation was determined by staining with 1 mg·mL^−1^ Alizarin red S solution (pH 5.5) after 21 days of induction.

For chondrocyte differentiation, cells were harvested and resuspended in α-MEM supplemented with 10% FBS and 1% penicillin/streptomycin. Droplets (10 µL) containing 10^6^ cells were placed in the middle of each well of a 24-well plate. After cells had adhered at 37 °C in 5% CO_2_ for 2 h, 500 mL chondrogenic medium supplemented with 1% insulin transferrin selenium solution (ITS, Sigma), 10 ng·mL^−1^ TGF-β (PeproTech), 100 nmol·L^−1^ dexamethasone (Sigma), 40 μg·mL^−1^ proline (MilliporeSigma), 50 μg·mL^−1^ L-ascorbic acid 2-phosphate (Sigma) and 1 mmol·L^−1^ sodium pyruvate (Thermo Fisher Scientific) was added. The medium was changed every 3 days. Micromass cultures were stained with Alcian blue at day 21.

For adipocyte differentiation, the medium contained solution A and solution B. Solution A, which contained 50 mmol·L^−1^ dexamethasone (Sigma), 100 mmol·L^−1^ rosiglitazone (Sigma), 500 nmol·L^−1^ IBMX (Sigma), and 10 mg·mL^−1^ insulin (Sigma), was incubated for 2 days, followed by solution B, which contained 10 mg·mL^−1^ insulin (Sigma), for 1 day. After 6 days (two rounds of induction), the cells were fixed in 4% PFA and stained with Oil Red O to detect lipids.

### scRNA-seq library construction, sequencing and data analysis

We constructed 3′ scRNA-seq libraries using a Chromium Controller (10X Genomics) and Single Cell 3′ Reagent Kit v2 (10X Genomics). Reverse transcription, cDNA amplification and library construction were performed on a T100 Thermal Cycler (Bio-Rad), followed by next-generation sequencing (Illumina NovaSeq, paired-end 150 bp). For data analysis, paired-end sequencing reads were processed using the Cell Ranger pipelines (version 3.0, 10X Genomics) with default parameters for sample demultiplexing, read mapping (mm10/GRCm38), barcode processing, single-cell transcript counting and matrix generation. The Seurat^41^ package was used for downstream analysis. To analyze *Prx1*^*Cre*^*;Z24*^*fl/fl*^ *Rosa26*^*Ai9*^ (2068 cells) and *Prx1*^*Cre*^*; Z24*^*fl/+*^ *Rosa26*^*Ai9*^ (2361 cells), scRNA-seq data were individually filtered by *“min.cells* = *3, min.features* = *200”*. With the top 22 PCs and 0.15 resolution, dimensions for all cells were reduced with uniform manifold approximation and projection (UMAP) and clustered into 5 subpopulations. To analyze *Prx1*^*Cre*^;*Rosa26*^*Ai9*^ (983 cells) and *Osx*^*Cre*^;*Rosa26*^*Ai9*^ (778 cells), scRNA-seq data were obtained after filtering as described above. The top 20 PCs and 0.14 resolution were used, while the other settings were the same as described above.

The Monocle 2 R package^42^ was used for pseudotemporal analysis. Clusters of BMSCs, PMSCs, chondrocytes and OBs identified by Seurat were imported into Monocle 2 to construct the CellDataSet object. The “DDRTree” method was used for dimensional reduction. We set the “max_components” to 8, and component 1 and component 3 were selected to generate the trajectory.

### RNA-seq library construction and data analysis

Total RNA was extracted from 1 000 cells following the manufacturer’s instructions and purified using an RNA purification kit (Tiangen, DP412). RNA libraries were constructed according to a modified SmartSeq2 protocol^43^ and then subjected to next-generation sequencing (Illumina, NovaSeq, paired-end 150 bp). For data analysis, the adapters were trimmed. Qualified data were mapped to the mouse reference genome (mm10) with STAR. HTSeq was used to summarize read counts, and duplicated reads were removed by UMI information (in Read2) to obtain gene counts. DESeq2 was used to identify differentially expressed genes (log2fold change > 0.25 and *P* value < 0.05). GO and KEGG enrichment analyses were performed with ClusterProfiler.^44^ GSEA was performed by using the MSigDB database.

### ATAC-seq library construction and data analysis

The transposition reaction was performed as instructed in the TruePrep DNA Library Prep Kit V2 (Vazyme), and the resulting DNA fragments were purified by a MinElute PCR Purification Kit (Qiagen). PCR amplification was performed by using the primers provided in the TruePrep Index Kit V2 (Vazyme) for 12-14 cycles (the number of PCR cycles was determined by qPCR as previously described^45^). DNA fragments with proper length (200–700 bp) were selected and purified using VAHTS DNA Clean Beads (Vazyme) and subjected to next-generation sequencing (Illumina NovaSeq system, paired-end 150 bp). For sequencing data analysis, the adapters were trimmed and aligned to the mm10 reference genome by Bowtie2. Reads mapped to mitochondrial DNA, and duplicates or unknown identities were eliminated by Sambamba. We performed peak calling wtih MACS2,^46^ summarized the read counts with bedtools, and calculated the differential peaks with DESeq2 (log2fold change > 0.25 and *P* value < 0.05). Motif enrichment in differential peaks was performed using HOMER.^47^

## Supplementary information

Supplementary Materials

Supplementary tableS3

Supplementary figures

## Data Availability

Single-cell RNA sequencing data, RNA sequencing data and ATAC sequencing data were deposited at GEO and can be obtained by GSE182540. The RNA sequencing data associated with bones after exercise were deposited at GEO and can be obtained by GSE207240.
