# Supplementary material for: Premature aging of skeletal stem/progenitor cells rather than osteoblasts causes bone loss with decreased mechanosensation
Source: Bone Res. 2023 Jul 5;11:35. doi: 10.1038/s41413-023-00269-6 (PMC10322990; doi:10.1038/s41413-023-00269-6)
Supplement: Supplementary file 1 — Supplementary Materials [file 41413_2023_269_MOESM1_ESM.docx]

**Table S1. 8-wk Wild-type Mouse Treadmill Running Protocol**

| **Start Age:** | **8-wk** |  | **Time Span:** | **5 weeks** |  |  |  |
| --- | --- | --- | --- | --- | --- | --- | --- |
|  | **Mon** | **Tue** | **Wed** | **Thu** | **Fri** | **Sat** | **Sun** |
| **1st week** | 12 m/min, 20 min | | 15 m/min, 30 min | | 18 m/min, 40 min | | Rest |
| **2nd-5th weeks** | 18 m/min, 50 min | | | | | |  |

**Table S2. 6-wk *Prx1^Cre^Z24^fl/fl^* and Littermate Treadmill Running Protocol**

| **Start Age:** | **6-wk** |  | **Time Span:** | **10 weeks** |  |  |  |
| --- | --- | --- | --- | --- | --- | --- | --- |
|  | **Mon** | **Tue** | **Wed** | **Thu** | **Fri** | **Sat** | **Sun** |
| **1st week** | 10 m/min, 20 min | | 12 m/min, 30 min | | 13 m/min, 40 min | | Rest |
| **2nd-10th weeks** | 13 m/min, 55 min | | | | | |  |

**Supplementary Materials**

**Supplementary Fig. 1. Generation and skeletal characterization of *Z24-KO first* mice.**

(**a**) Structure of the genomic *Zmpste24* locus, Z24-KO first targeting vector after homologous recombination. ex: exon; int: intron; orange box: synthetic cassette replacing partial intron 5 sequence. Half arrows, forward primer (Z24-F), reverse primers (Z24-WT-R, Z24-KO-R); Right, PCR genotyping wild-type allele, 131 bp; ko first allele, 319 bp. (**b**) Gene expression of Zmpste24 in liver, lung and bone from *Zmpste24-KO first* mice (n=4) and WT littermate controls (n=4). (**c**) Western blotting analysis of Lamin A, Prelamin A and Lamin C expression in liver, lung and bone from *Zmpste24-KO first* mice and WT littermate controls. (**d**) Body weight curve of *Z24-KO first* male mice and their littermate controls. n=5 per group. (**e**) Whole mount skeleton staining of postnatal Day 1 *Z24-KO first* mice and littermate controls by Alizarin red S and Alcian blue. Scale bar, 5 mm. (**f**) Alizarin Red S- and Alcian Blue-stained head and leg of *Z24-KO first* mice and littermate controls. Scale bar, 3 mm. (**g**) Measurements of skull length, skull width, femur length and tibia length. n=4 per group. (**h**) Safranin O staining of postnatal Day 3 *Z24-KO first* mice and littermate controls. Scale bar, 100 μm. (**i**) Representative microCT images of 8- and 16-week-old *Z24-KO first* mice (n=4) and WT littermate controls (n=4). (**j-n**) Quantitative analyses of trabecular bone volume/total volume (**j**), trabecular thickness (**k**), trabecular number (**l**), trabecular spacing (**m**) and cortical thickness (**n**) in the femur metaphysis of 8- and 16-week-old *Z24-KO first* mice (n=4) and WT littermate controls (n=4). (**o**) Tartrate-resistant acid phosphatase (TRAP) staining of femurs of 8- and 12-week-old *Z24-KO first* mice and littermate controls. Scale bar, 50 μm. (**p**) Quantification of TRAP staining by osteoclast surface per bone surface (Oc.S/BS). The statistical significance of differences was assessed using two-tailed Student’s paired *t* test (**P* <0.05, ***P* <0.01, ****P* <0.001 and *****P* <0.0001).

**Supplementary Fig. 2. Generation and skeletal development characterization of *Z24-CKO* mice.**

(**a**) Structure of the genome of *Zmpste24^F/F^* mice. *Z24-KO first* mice were mated to *Flip* mice to obtain *Z24^F/+^* offspring in which the synthetic cassette between 2 Frt sites was deleted (length: 6951 bp). Half arrows, forward primer (Z24-F), reverse primer (Z24F/F-R); Right, PCR genotyping wild-type allele, 500 bp; Z24^fl/fl^, 607 bp. (**b**) Relative mRNA levels of Z24 in bone tissue of Z24-CKO mice were quantified by RT‒PCR. The statistical significance of differences was assessed using two-tailed Student’s paired *t* test (**P* <0.05, ***P* <0.01, ****P* <0.001 and *****P* <0.0001). (**c**) Body weight curves of *Prx1^Cre^;Z24^fl/fl^* mice and littermate controls. n=3 per group. (**d**) Whole mount skeleton staining of *Prx1^Cre^;Z24^fl/fl^* mice and littermate controls on postnatal Day 1 by Alizarin red S and Alcian blue. Scale bar, 5 mm. (**e**) Alizarin Red S- and Alcian Blue-stained head and leg of *Prx1^Cre^;Z24^fl/fl^* mice and littermate controls. Scale bar, 3 mm. (**f**) Measurements of skull length, skull width, femur length and tibia length of *Prx1^Cre^;Z24^fl/fl^* mice and littermate controls. n=4 per group. (**g**) Safranin O staining of *Prx1^Cre^;Z24^fl/fl^* mice and littermate controls on postnatal Day 3. Scale bar, 100 μm. The statistical significance of differences was assessed using two-tailed Student’s paired *t* test (**P* <0.05, ***P* <0.01, ****P* <0.001 and *****P* <0.0001). (**h**) TRAP staining of femurs of 8- and 16-week-old *Prx1^Cre^;Z24^fl/fl^* mice and littermate controls. Scale bar, 50 μm. (**i**) Quantification of TRAP staining by osteoclast surface per bone surface (Oc.S/BS). The statistical significance of differences was assessed using two-tailed Student’s paired *t* test (**P* <0.05, ***P* <0.01, ****P* <0.001 and *****P* <0.0001).

**Supplementary Fig. 3. gpSSPCs and pSSPCs have distinct cell fates.**

(**a**) State results from pseudotime analysis of 4073 total whole bone *Prx1^Cre+^* cells (except for the pericyte cluster). (**b**) Branch kinetic curves of markers of adipocytes (Adipoq and Lpl), osteoblasts (Col1a1) and stromal cells (Lepr and Kitl). (**c**) UMAP plots of PMSC and chondrocyte clusters of 1634 cell scRNA profiles from 8-week-old *Prx1^Cre^;Rosa26^Ai9^* mice and *Osx^Cre^;Rosa26^Ai9^* mice showing the expression levels and distribution of osteochondrogenic skeletal stem cell (ocSSC) and perivascular SSC (pvSSC) top genes as shown by Ambrosi, T. H ^29^.

**Supplementary Fig. 4. Apoptosis pathway and extracellular matrix degradation in prematurely aged pSSPCs.**

**(a)** Normalized ATAC-seq signal intensity around the transcription start site (TSS). n=3 per group. Right: histograms show genome-wide chromatin accessibility at the TSS. **(b)** GO terms enriched for significantly different genes identified by RNA-seq. Selected GO terms with significant *P* values are shown. **(c-e)** GSEA of the expression of Reactome Apoptosis by CDKN1A via TP53 (**c**), Invasion inhibited by Ascites DN (**d**) and Uterine fibroid up (**e**) of pSSPCs. n=3 per group. **(f, g)** Venn diagram (**f**) showing the overlap of genes with open chromatin in close proximity and transcripts and selected ATAC-seq signal tracks (**g**) that were differentially upregulated in pSSPCs. The number of genes near peaks that were accessible and significantly differentially expressed genes is shown. **(h, i)** Venn diagram (**h**) showing the overlap of genes with open chromatin in close proximity and transcripts and selected ATAC-seq signal tracks (**i**) that were differentially downregulated in pSSPCs. The number of genes near peaks that were accessible and significantly differentially expressed genes is shown. **(j)** EM images of the periosteum. Left: EM methodology. Consecutive periosteum, vertical thin sections analyzed by toluidine blue. Scale bars, 1 μm.

**Supplementary Fig. 5. Physical exercise increases bone mass and SSPC number.**

**(a)** Schematic showing the treadmill experimental strategy. **(b)** Representative microCT images of femurs (n=3). **(c-g)** Quantitative analyses of trabecular bone volume/total volume (BV/TV) (**c**), trabecular thickness (Tb.Th) (**d**), trabecular spacing (Tb.Sp) (**e**), trabecular number (Tb.N) (**f**) and cortical thickness (Ct.Th) (**g**) in the femurs. The statistical significance of differences was assessed using two-tailed Student’s paired *t* test (**P* <0.05, ***P* <0.01, ****P* <0.001 and *****P* <0.0001). **(h)** Fluorescence images of CD73 and Col2 in the growth plate from mice that exercised for 5 weeks at 13 weeks of age. Sedentary littermates were used as controls. Scale bar, 25 μm.

**Supplementary Fig. 6. Combined analysis of bulk RNA-seq of premature gpSSPCs and bones after exercise.**

**(a, b)** Venn diagram showing the overlap of transcripts **(a)** that are upregulated in prematurely aged gpSSPCs and downregulated in bones after exercise and the overlap of transcripts **(b)** that were downregulated in prematurely aged gpSSPC and upregulated in bones after 5 weeks of exercise. **(c, d)** Heatmap of gene expression of overlapping transcripts **(c)** shown as **(a)** and transcripts **(d)** shown as **(b)**. **(e, f)** GO terms enriched for overlapping genes shown in **(a, b)**. Selected GO terms with significant *P* values are shown.

**Supplementary Fig. 7. Mechanical stimuli reverse cellular apoptosis in bones after exercise.**

**(a-c)** Gene expression of *p16/Cdkn2a* **(a)**, *p21/Cdkn1a* **(b)** and *Col2a1* **(c)** in bone after bone marrow was flushed from 16-week-old exercised and sedentary *Prx1^Cre^; Z24^fl/fl^* (CKO) and *Z24^fl/fl^* (WT) mice. (**d**) Proportion of cleaved Caspase 3^+^ cells in tissue sections from *Prx1^Cre^; Z24^fl/fl^* mice and control littermates after exercise. The statistical significance of differences was assessed using two-tailed Student’s paired *t* test (**P* <0.05, ***P* <0.01, ****P* <0.001 and *****P* <0.0001).
