# Supplementary figures and images for: Premature aging of skeletal stem/progenitor cells rather than osteoblasts causes bone loss with decreased mechanosensation
Source: Bone Res. 2023 Jul 5;11:35. doi: 10.1038/s41413-023-00269-6 (PMC10322990; doi:10.1038/s41413-023-00269-6)

**Fig S1 a**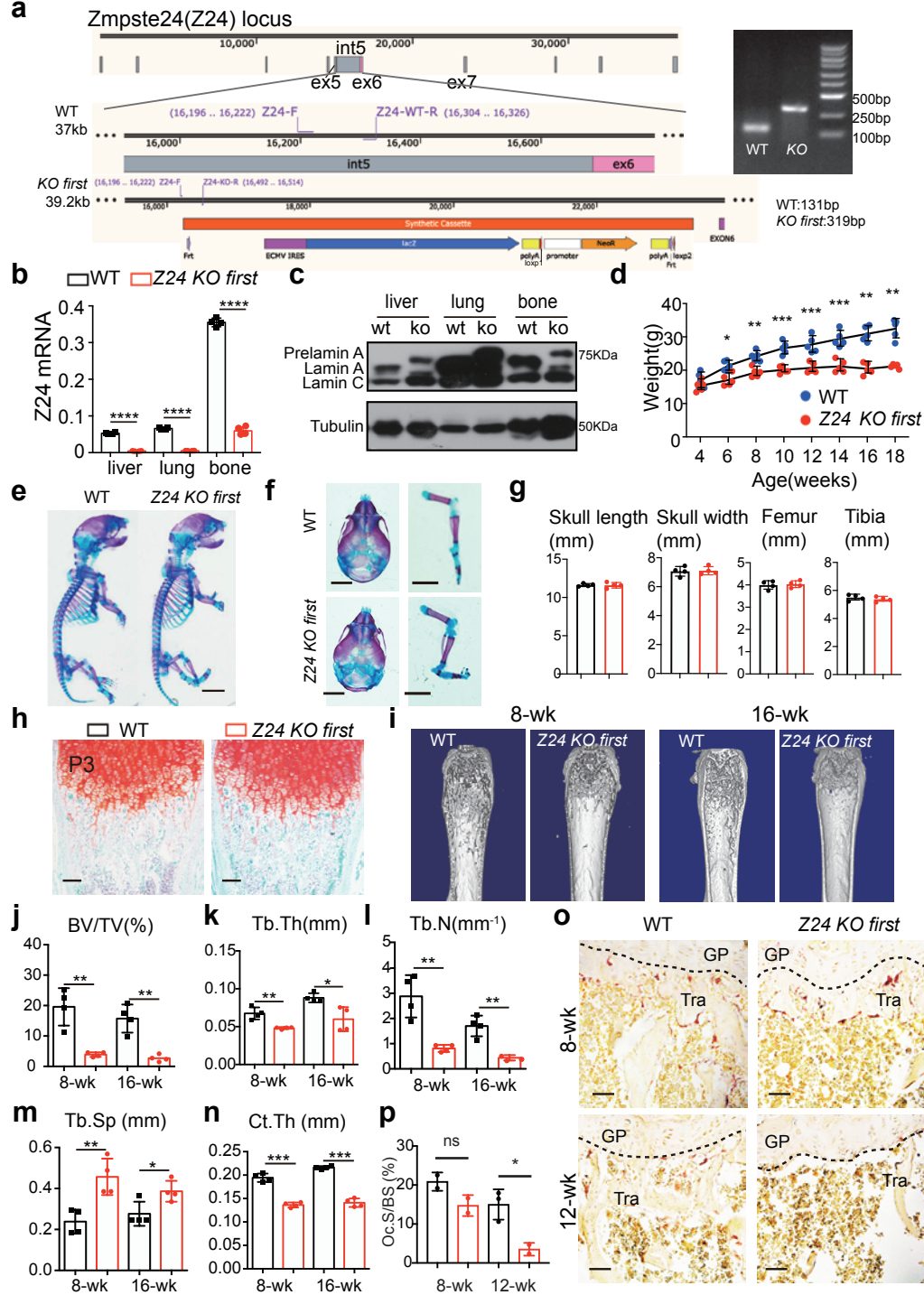

Fig S2

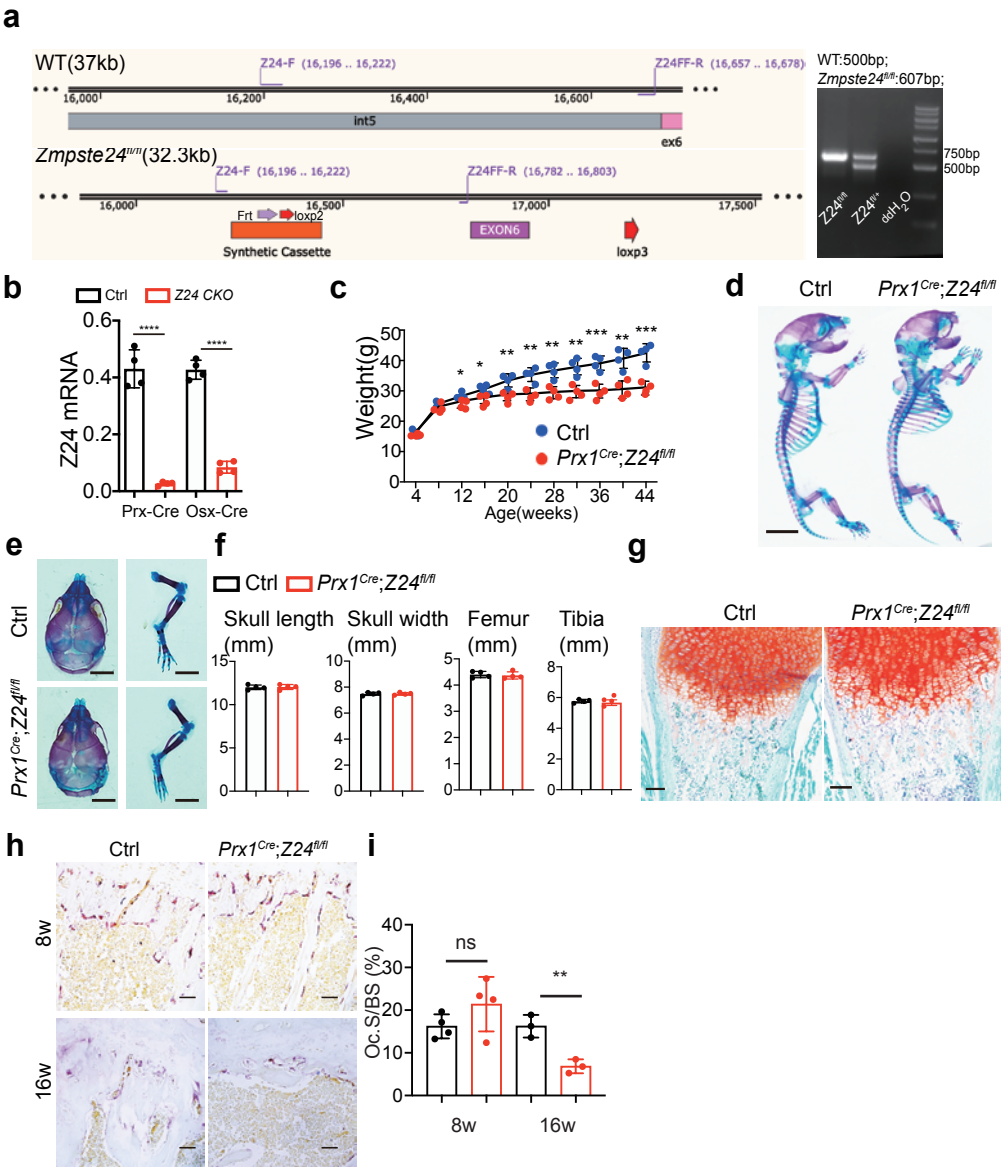

Fig S3

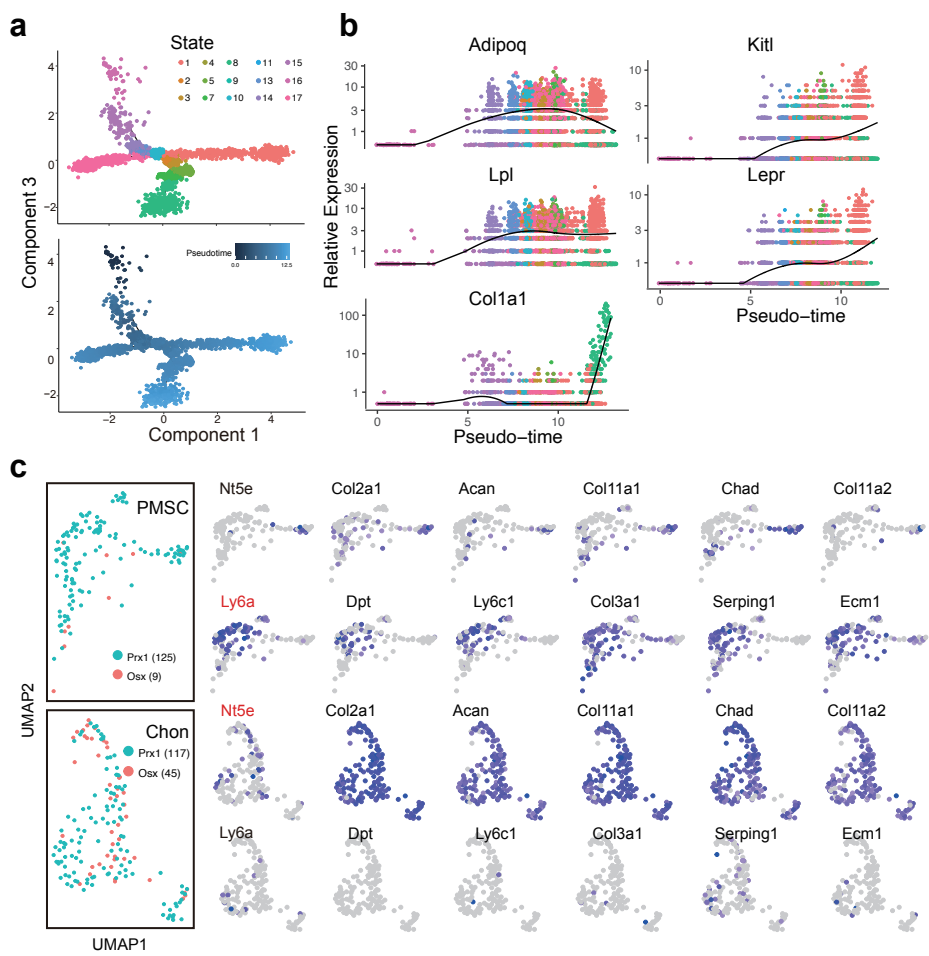

**Fig S4**

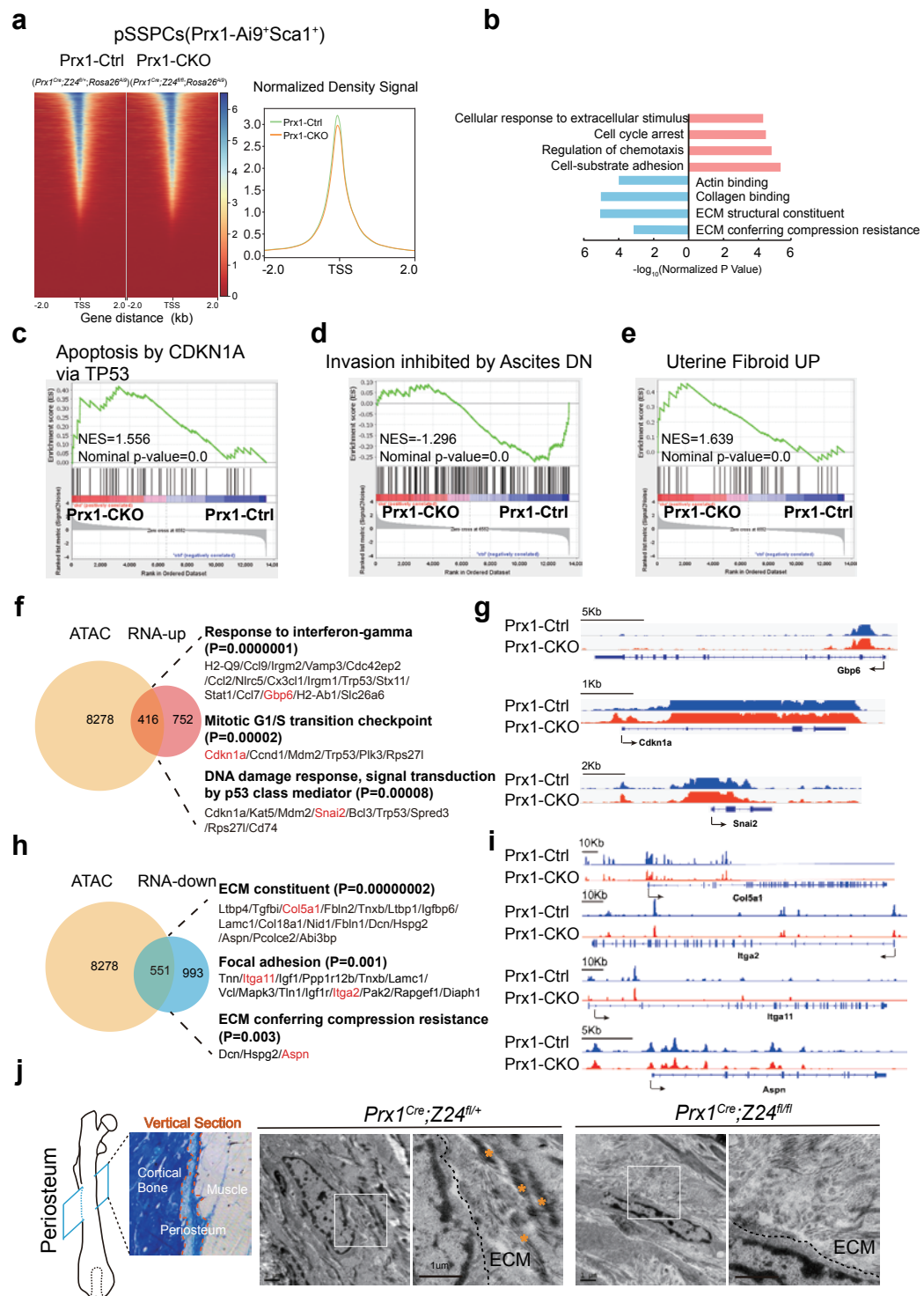

Fig S5

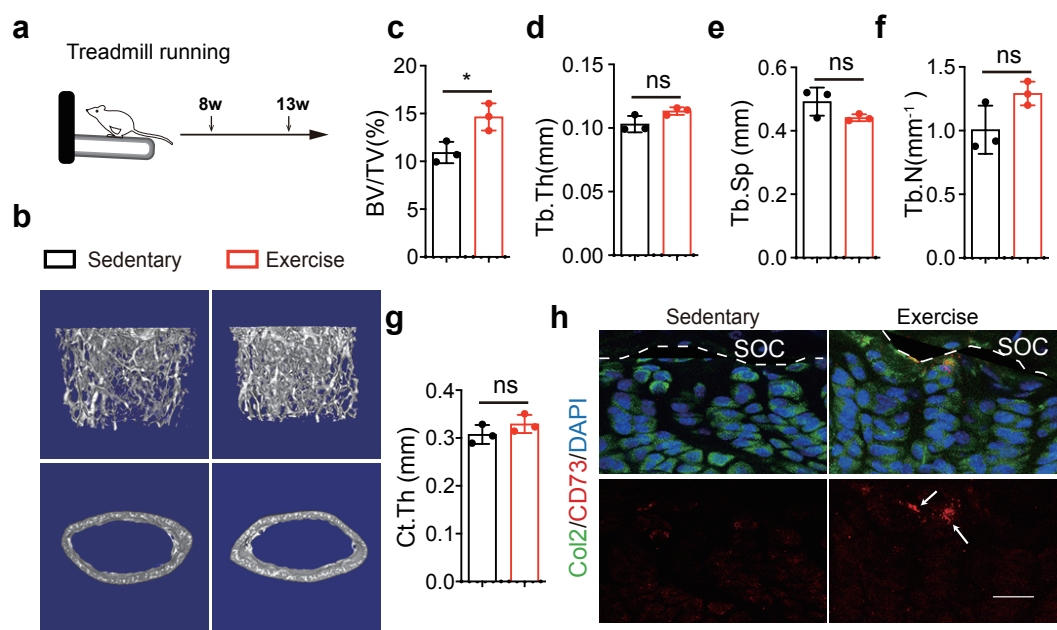

Fig S6

a

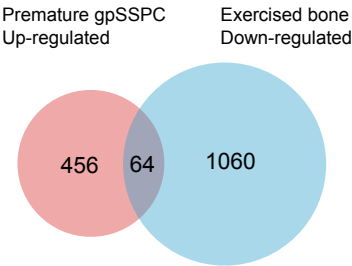

b

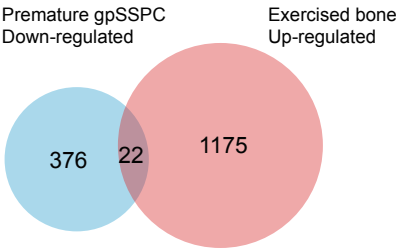

d

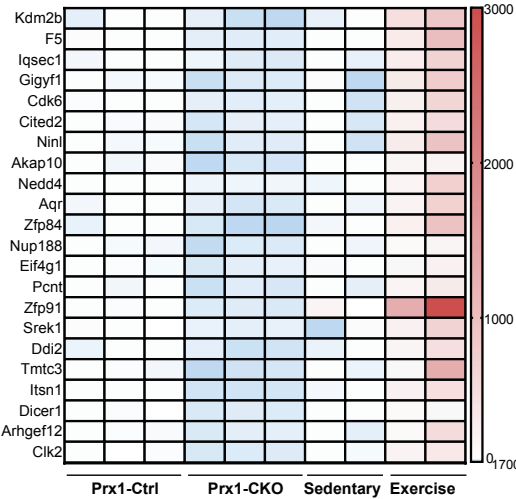

e

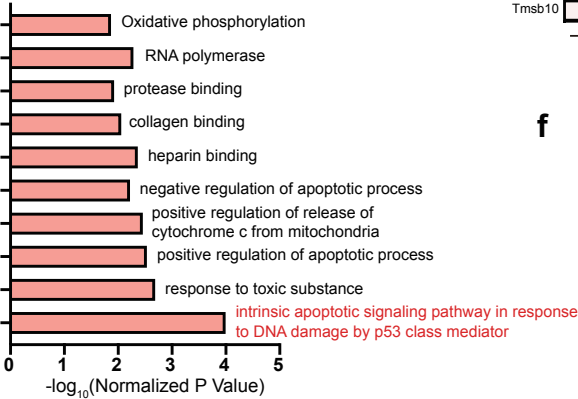

c

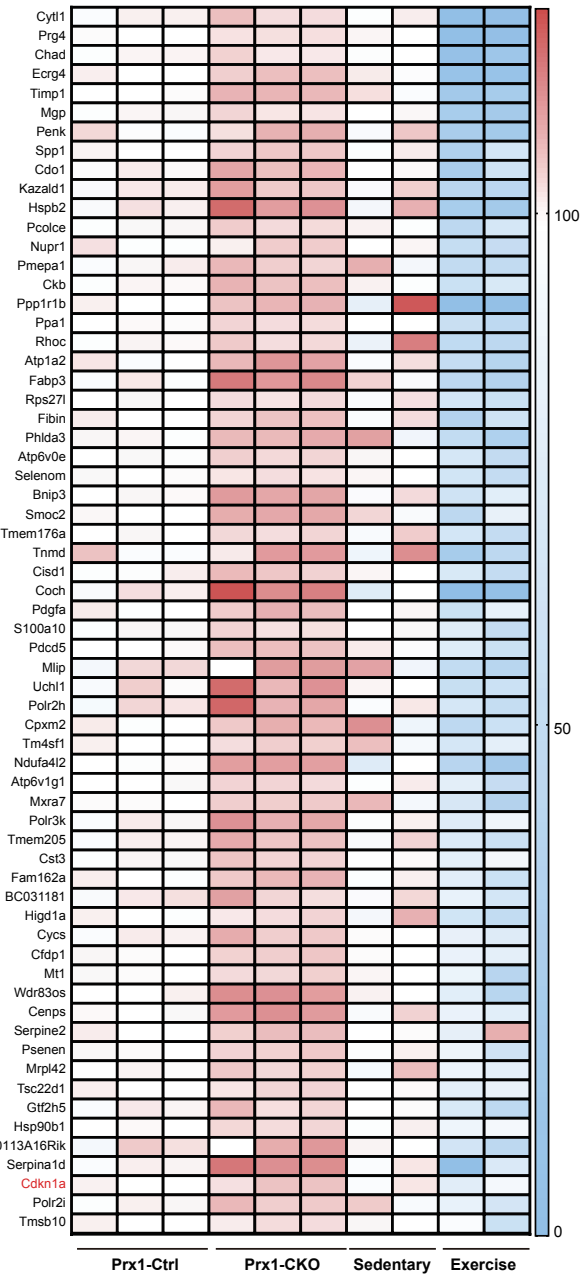

f

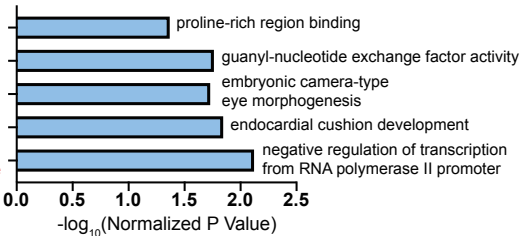

Fig S7

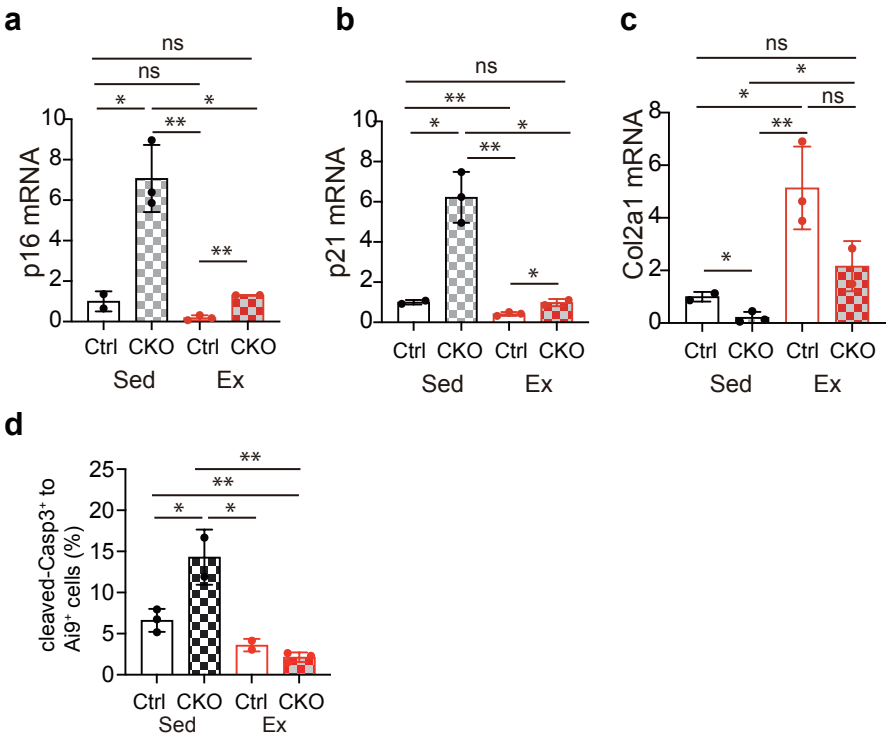

Supplement: Supplementary file 3 — Supplementary figures [file 41413_2023_269_MOESM3_ESM.pdf]
